# Supplementary material for: Root Na+ Content Negatively Correlated to Salt Tolerance Determines the Salt Tolerance of Brassica napus L. Inbred Seedlings
Source: Plants (Basel). 2022 Mar 29;11(7):906. doi: 10.3390/plants11070906 (PMC9002931; doi:10.3390/plants11070906)
Supplement: Supplementary file 1 [file plants-11-00906-s001.zip › Supplementary Materials S1.pdf]

Supplementary Materials S1. Absolute and relative values of the *B. napus* growth indexes

Salt tolerance levels	No. of inbred lines	NaCl levels	Values	Fresh weight of shoots (mg/ plant)	Fresh weight of roots (mg/ plant)	Total fresh weight (mg/plant)	Dry weight of shoots (mg/ plant)	Dry weight of roots (mg/ plant)	Total dry weight (mg/plant)	Shoot length (cm/plant)	Root length (cm/plant)	Dry weight of shoots/Fresh weight of shoots	Dry weight of roots/Fresh weight of roots
HSS (1)	557	0	Absolute value	31.49 ± 1.08	13.25 ± 1.15	44.74 ± 2.21	3.02 ± 0.07	0.74 ± 0.11	3.76 ± 0.07	2.73 ± 0.72	11.96 ± 3.17	0.10 ± 4.83E-03	0.06 ± 3.30E-03
			Relative value	1.00	1.00	1.00	1.00	1.00	1.00	1.00	1.00	1.00	1.00
		50	Absolute value	25.28 ± 1.28	11.91 ± 1.55	37.19 ± 2.28	3.01 ± 0.05	0.68 ± 0.10	3.69 ± 0.14	1.96 ± 0.53	10.40 ± 2.73	0.12 ± 4.33E-03	0.06 ± 1.68E-03
			Relative value	0.80	0.90	0.83	1.00	0.93	0.98	0.72	0.87	1.24	1.03
		100	Absolute value	18.99 ± 1.21	9.06 ± 1.29	28.05 ± 0.81	2.77 ± 0.17	0.57 ± 0.10	3.34 ± 0.11	1.42 ± 0.39	8.06 ± 1.84	0.15 ± 1.42E-03	0.06 ± 3.10E-03
			Relative value	0.60	0.68	0.63	0.92	0.77	0.89	0.52	0.67	1.52	1.13
		150	Absolute value	11.07 ± 0.44	6.22 ± 1.23	17.29 ± 0.82	1.68 ± 0.13	0.43 ± 0.04	2.10 ± 0.12	1.19 ± 0.36	4.76 ± 1.63	0.15 ± 0.01	0.07 ± 0.01
			Relative value	0.35	0.47	0.39	0.56	0.58	0.56	0.44	0.40	1.58	1.25
		200	Absolute value	/	4.69 ± 0.05	/	/	0.33 ± 0.02	/	/	3.35 ± 1.08	/	0.07 ± 3.06E-03
			Relative value	/	0.35	/	/	0.45	/	/	0.28	/	1.28
	611	0	Absolute value	24.91 ± 2.48	13.10 ± 0.39	38.01 ± 2.87	2.25 ± 0.12	0.84 ± 0.08	3.09 ± 0.20	3.84 ± 0.63	8.89 ± 2.31	0.09 ± 4.52E-03	0.06 ± 4.77E-03
			Relative value	1.00	1.00	1.00	1.00	1.00	1.00	1.00	1.00	1.00	1.00
		50	Absolute value	18.74 ± 2.89	9.15 ± 0.60	27.89 ± 2.84	2.01 ± 0.16	0.63 ± 0.05	2.63 ± 0.12	2.57 ± 0.52	7.20 ± 2.81	0.11 ± 0.01	0.07 ± 9.30E-04
			Relative value	0.75	0.70	0.73	0.89	0.74	0.85	0.67	0.81	1.20	1.06
		100	Absolute value	13.41 ± 2.10	8.21 ± 1.58	21.62 ± 2.32	1.81 ± 0.23	0.57 ± 0.07	2.38 ± 0.17	2.06 ± 0.43	7.11 ± 2.21	0.14 ± 0.03	0.07 ± 0.01
			Relative value	0.54	0.63	0.57	0.81	0.67	0.77	0.54	0.80	1.52	1.09
		150	Absolute value	9.35 ± 0.48	5.40 ± 0.51	14.74 ± 0.98	1.50 ± 0.26	0.42 ± 0.08	1.92 ± 0.34	1.31 ± 0.29	4.96 ± 1.71	0.16 ± 0.02	0.08 ± 0.01
			Relative value	0.38	0.41	0.39	0.67	0.49	0.62	0.34	0.56	1.77	1.19
		200	Absolute value	9.34 ± 1.40	3.96 ± 0.84	13.31 ± 1.88	1.57 ± 0.20	0.34 ± 0.11	1.92 ± 0.24	1.24 ± 0.14	3.59 ± 1.07	0.17 ± 0.05	0.09 ± 0.01
			Relative value	0.38	0.30	0.35	0.70	0.41	0.62	0.32	0.40	1.91	1.33
	668	0	Absolute value	30.03 ± 0.58	13.71 ± 1.01	43.74 ± 1.54	3.19 ± 0.07	0.77 ± 0.08	3.95 ± 0.08	2.83 ± 0.53	8.66 ± 1.99	0.11 ± 2.09E-03	0.06 ± 3.23E-03
			Relative value	1.00	1.00	1.00	1.00	1.00	1.00	1.00	1.00	1.00	1.00
		50	Absolute value	22.60 ± 0.93	13.92 ± 0.77	36.52 ± 0.93	2.83 ± 0.14	0.79 ± 0.10	3.61 ± 0.22	1.89 ± 0.48	7.82 ± 3.69	0.13 ± 3.74E-03	0.06 ± 0.01
			Relative value	0.75	1.02	0.83	0.89	1.03	0.91	0.67	0.90	1.18	1.02
		100	Absolute value	21.47 ± 1.71	9.66 ± 1.48	31.13 ± 3.05	3.03 ± 0.29	0.63 ± 0.07	3.66 ± 0.34	1.53 ± 0.36	7.00 ± 2.26	0.14 ± 4.43E-03	0.07 ± 2.80E-03
			Relative value	0.72	0.70	0.71	0.95	0.83	0.93	0.54	0.81	1.33	1.18
		150	Absolute value	14.54 ± 2.22	6.75 ± 0.19	21.30 ± 2.06	2.20 ± 0.16	0.45 ± 0.03	2.65 ± 0.14	1.14 ± 0.22	5.16 ± 1.73	0.15 ± 0.01	0.07 ± 0.01
			Relative value	0.48	0.49	0.49	0.69	0.59	0.67	0.40	0.60	1.44	1.20
		200	Absolute value	12.87 ± 0.83	4.93 ± 0.52	17.79 ± 0.43	1.69 ± 0.15	0.31 ± 0.07	2.00 ± 0.10	0.86 ± 0.09	2.31 ± 0.80	0.13 ± 0.01	0.06 ± 0.01
			Relative value	0.43	0.36	0.41	0.53	0.40	0.51	0.31	0.27	1.24	1.13
	703	0	Absolute value	30.61 ± 1.24	15.96 ± 0.50	46.58 ± 1.00	3.20 ± 0.09	0.92 ± 0.05	4.12 ± 0.09	2.97 ± 0.52	10.47 ± 2.66	0.10 ± 1.72E-03	0.06 ± 3.68E-03
			Relative value	1.00	1.00	1.00	1.00	1.00	1.00	1.00	1.00	1.00	1.00
		50	Absolute value	23.01 ± 1.59	11.81 ± 0.64	34.83 ± 1.12	2.77 ± 0.20	0.66 ± 0.05	3.43 ± 0.15	2.09 ± 0.53	9.05 ± 3.48	0.12 ± 3.71E-03	0.06 ± 1.93E-03
			Relative value	0.75	0.74	0.75	0.87	0.72	0.83	0.71	0.86	1.15	0.97
		100	Absolute value	22.34 ± 3.72	9.19 ± 1.21	31.53 ± 4.84	3.04 ± 0.43	0.60 ± 0.06	3.64 ± 0.46	1.56 ± 0.52	5.64 ± 3.00	0.14 ± 4.41E-03	0.07 ± 3.55E-03
			Relative value	0.73	0.58	0.68	0.95	0.66	0.88	0.52	0.54	1.30	1.15
		150	Absolute value	15.71 ± 3.67	4.65 ± 0.24	20.36 ± 3.76	2.87 ± 0.35	0.34 ± 0.01	3.21 ± 0.34	1.17 ± 0.19	3.93 ± 1.63	0.19 ± 0.03	0.07 ± 3.87E-03
			Relative value	0.51	0.29	0.44	0.90	0.37	0.78	0.40	0.38	1.79	1.27
		200	Absolute value	11.05 ± 1.85	4.67 ± 0.25	15.72 ± 1.74	2.17 ± 0.22	0.28 ± 0.02	2.45 ± 0.20	0.91 ± 0.20	2.56 ± 1.09	0.20 ± 0.02	0.06 ± 4.01E-03
			Relative value	0.36	0.29	0.34	0.68	0.31	0.59	0.31	0.24	1.89	1.05
Average		0	Absolute value	29.26 ± 2.96	14.01 ± 1.33	43.27 ± 3.69	2.91 ± 0.45	0.82 ± 0.08	3.73 ± 0.45	3.09 ± 0.51	9.99 ± 1.53	0.10 ± 0.01	0.06 ± 4.06E-03
			Relative value	1.00	1.00	1.00	1.00	1.00	1.00	1.00	1.00	1.00	1.00

Supplementary Materials S1 continued

		50	Absolute value	22.41 ± 2.72	11.70 ± 1.96	34.11 ± 4.26	2.65 ± 0.44	0.69 ± 0.07	3.34 ± 0.49	2.13 ± 0.30	8.62 ± 1.42	0.12 ± 0.01	0.06 ± 0.01
			Relative value	0.76	0.84	0.79	0.91	0.85	0.90	0.69	0.86	1.19	1.02
		100	Absolute value	19.05 ± 4.02	9.03 ± 0.61	28.08 ± 4.58	2.66 ± 0.58	0.59 ± 0.03	3.26 ± 0.60	1.64 ± 0.28	6.95 ± 1.00	0.14 ± 4.35E-03	0.07 ± 2.86E-03
			Relative value	0.65	0.65	0.65	0.91	0.73	0.87	0.53	0.71	1.42	1.14
		150	Absolute value	12.67 ± 2.96	5.75 ± 0.93	18.42 ± 2.99	2.06 ± 0.61	0.41 ± 0.05	2.47 ± 0.58	1.20 ± 0.07	4.70 ± 0.54	0.16 ± 0.02	0.07 ± 4.20E-03
			Relative value	0.43	0.42	0.42	0.70	0.51	0.66	0.39	0.48	1.64	1.23
		200	Absolute value	11.09 ± 1.76	4.56 ± 0.42	15.61 ± 2.25	1.81 ± 0.31	0.32 ± 0.03	2.12 ± 0.29	1.01 ± 0.17	2.95 ± 0.61	0.17 ± 0.03	0.07 ± 0.01
			Relative value	0.39	0.33	0.36	0.64	0.39	0.57	0.31	0.30	1.68	1.20
SS (2)	40	0	Absolute value	30.53 ± 3.53	14.54 ± 1.66	45.07 ± 5.15	3.10 ± 0.18	0.83 ± 0.05	3.93 ± 0.14	3.27 ± 0.29	10.16 ± 1.40	0.10 ± 0.01	0.06 ± 0.01
			Relative value	1.00	1.00	1.00	1.00	1.00	1.00	1.00	1.00	1.00	1.00
		50	Absolute value	28.29 ± 1.39	13.42 ± 0.41	41.71 ± 0.97	3.16 ± 0.18	0.76 ± 0.03	3.92 ± 0.15	2.67 ± 0.43	8.64 ± 2.29	0.11 ± 9.98E-04	0.06 ± 1.47E-03
			Relative value	0.93	0.92	0.93	1.02	0.91	1.00	0.82	0.85	1.10	0.97
		100	Absolute value	19.88 ± 0.63	11.15 ± 0.38	31.03 ± 1.00	2.77 ± 0.11	0.71 ± 0.04	3.48 ± 0.12	2.28 ± 0.25	7.10 ± 1.99	0.14 ± 0.01	0.06 ± 1.26E-03
			Relative value	0.65	0.77	0.69	0.89	0.86	0.89	0.70	0.70	1.37	1.10
		150	Absolute value	15.23 ± 2.97	9.79 ± 0.54	25.02 ± 3.27	2.63 ± 0.13	0.63 ± 0.05	3.26 ± 0.09	2.00 ± 0.34	4.98 ± 1.45	0.18 ± 0.02	0.06 ± 0.01
			Relative value	0.50	0.67	0.56	0.85	0.76	0.83	0.61	0.49	1.72	1.12
		200	Absolute value	12.70 ± 0.41	5.71 ± 0.77	18.41 ± 0.83	2.18 ± 0.06	0.39 ± 0.01	2.57 ± 0.07	1.72 ± 0.14	3.82 ± 0.80	0.17 ± 8.93E-04	0.07 ± 0.01
			Relative value	0.42	0.39	0.41	0.71	0.46	0.65	0.53	0.38	1.69	1.18
	74	0	Absolute value	31.55 ± 1.82	19.45 ± 1.07	51.00 ± 2.24	3.44 ± 0.13	1.03 ± 0.10	4.47 ± 0.22	3.14 ± 0.24	8.68 ± 1.93	0.11 ± 2.92E-03	0.05 ± 0.01
			Relative value	1.00	1.00	1.00	1.00	1.00	1.00	1.00	1.00	1.00	1.00
		50	Absolute value	29.29 ± 1.81	14.52 ± 2.21	43.81 ± 3.31	3.19 ± 0.21	0.86 ± 0.11	4.05 ± 0.28	2.83 ± 0.38	6.50 ± 1.46	0.11 ± 1.12E-03	0.06 ± 1.60E-03
			Relative value	0.93	0.75	0.86	0.93	0.83	0.91	0.90	0.75	1.00	1.12
		100	Absolute value	20.64 ± 1.38	8.65 ± 0.24	29.29 ± 1.41	2.56 ± 0.12	0.53 ± 0.03	3.08 ± 0.15	2.64 ± 0.38	6.14 ± 1.38	0.12 ± 3.06E-03	0.06 ± 3.94E-03
			Relative value	0.65	0.44	0.57	0.74	0.51	0.69	0.84	0.71	1.14	1.15
		150	Absolute value	15.58 ± 3.11	7.97 ± 0.29	23.55 ± 3.39	2.27 ± 0.32	0.53 ± 0.02	2.80 ± 0.32	1.99 ± 0.30	5.41 ± 0.76	0.15 ± 0.01	0.07 ± 3.10E-03
			Relative value	0.49	0.41	0.46	0.66	0.51	0.63	0.64	0.62	1.35	1.25
		200	Absolute value	15.04 ± 1.32	5.68 ± 0.94	20.72 ± 1.23	2.29 ± 0.24	0.29 ± 0.02	2.58 ± 0.23	1.61 ± 0.14	3.66 ± 0.67	0.15 ± 0.01	0.05 ± 0.01
			Relative value	0.48	0.29	0.41	0.67	0.28	0.58	0.51	0.42	1.39	0.97
	277	0	Absolute value	35.63 ± 1.49	13.13 ± 0.90	48.76 ± 2.39	3.70 ± 0.30	0.72 ± 0.08	4.42 ± 0.37	3.77 ± 0.63	10.03 ± 2.19	0.10 ± 0.01	0.05 ± 2.32E-03
			Relative value	1.00	1.00	1.00	1.00	1.00	1.00	1.00	1.00	1.00	1.00
		50	Absolute value	28.48 ± 0.39	11.80 ± 1.41	40.28 ± 1.43	3.54 ± 0.07	0.67 ± 0.05	4.21 ± 0.08	2.75 ± 0.51	9.43 ± 1.64	0.12 ± 8.86E-04	0.06 ± 2.17E-03
			Relative value	0.80	0.90	0.83	0.96	0.93	0.95	0.73	0.94	1.20	1.04
		100	Absolute value	25.27 ± 1.49	8.91 ± 0.97	34.18 ± 2.17	3.41 ± 0.06	0.55 ± 0.05	3.95 ± 0.10	2.01 ± 0.33	6.52 ± 2.13	0.14 ± 0.01	0.06 ± 3.67E-03
			Relative value	0.71	0.68	0.70	0.92	0.76	0.90	0.53	0.65	1.30	1.13
		150	Absolute value	18.24 ± 3.28	5.45 ± 1.35	23.69 ± 4.58	3.09 ± 0.59	0.42 ± 0.17	3.51 ± 0.75	1.57 ± 0.22	5.54 ± 1.31	0.17 ± 2.45E-03	0.07 ± 0.01
			Relative value	0.51	0.42	0.49	0.83	0.59	0.79	0.42	0.55	1.63	1.37
		200	Absolute value	17.29 ± 1.99	4.97 ± 0.67	22.26 ± 2.05	2.94 ± 0.42	0.31 ± 0.07	3.25 ± 0.41	1.40 ± 0.17	3.44 ± 0.98	0.17 ± 4.43E-03	0.06 ± 4.94E-03
			Relative value	0.49	0.38	0.46	0.79	0.43	0.74	0.37	0.34	1.63	1.14
	323	0	Absolute value	34.89 ± 2.44	15.26 ± 1.23	50.15 ± 3.65	3.23 ± 0.34	0.87 ± 0.11	4.11 ± 0.43	3.86 ± 0.34	9.96 ± 2.85	0.09 ± 3.25E-03	0.06 ± 3.93E-03
			Relative value	1.00	1.00	1.00	1.00	1.00	1.00	1.00	1.00	1.00	1.00
		50	Absolute value	28.96 ± 2.94	15.42 ± 1.87	44.39 ± 2.27	3.85 ± 0.23	0.92 ± 0.10	4.77 ± 0.21	2.63 ± 0.32	7.59 ± 1.37	0.13 ± 0.01	0.06 ± 7.83E-04
			Relative value	0.83	1.01	0.89	1.19	1.06	1.16	0.68	0.76	1.44	1.05
		100	Absolute value	22.33 ± 2.14	11.02 ± 0.27	33.35 ± 2.41	3.05 ± 0.38	0.74 ± 0.04	3.79 ± 0.38	2.03 ± 0.38	5.17 ± 1.14	0.14 ± 4.13E-03	0.07 ± 4.10E-03
			Relative value	0.64	0.72	0.67	0.94	0.84	0.92	0.52	0.52	1.47	1.17
		150	Absolute value	15.85 ± 0.90	7.45 ± 1.03	23.30 ± 0.63	2.78 ± 0.13	0.54 ± 0.05	3.32 ± 0.15	1.68 ± 0.23	4.24 ± 1.05	0.18 ± 2.64E-03	0.07 ± 0.01
			Relative value	0.45	0.49	0.46	0.86	0.62	0.81	0.44	0.43	1.90	1.29

Supplementary Materials S1 continued

		200	Absolute value	20.28 ± 3.19	6.26 ± 0.48	26.54 ± 3.65	3.10 ± 0.53	0.41 ± 0.04	3.51 ± 0.57	1.58 ± 0.13	2.64 ± 0.47	0.15 ± 3.66E-03	0.07 ± 2.36E-03
			Relative value	0.58	0.41	0.53	0.96	0.47	0.85	0.41	0.27	1.65	1.15
	Average	0	Absolute value	33.15 ± 2.49	15.60 ± 2.72	48.75 ± 2.62	3.37 ± 0.26	0.86 ± 0.13	4.23 ± 0.26	3.51 ± 0.36	9.71 ± 0.69	0.10 ± 0.01	0.06 ± 2.39E-03
			Relative value	1.00	1.00	1.00	1.00	1.00	1.00	1.00	1.00	1.00	1.00
		50	Absolute value	28.76 ± 0.45	13.79 ± 1.56	42.55 ± 1.90	3.44 ± 0.32	0.80 ± 0.11	4.24 ± 0.37	2.72 ± 0.09	8.04 ± 1.27	0.12 ± 0.01	0.06 ± 1.71E-03
			Relative value	0.87	0.89	0.87	1.02	0.93	1.00	0.78	0.83	1.18	1.05
		100	Absolute value	22.03 ± 2.39	9.93 ± 1.34	31.96 ± 2.22	2.95 ± 0.37	0.63 ± 0.11	3.58 ± 0.38	2.24 ± 0.30	6.23 ± 0.81	0.13 ± 0.01	0.06 ± 2.71E-03
			Relative value	0.66	0.65	0.66	0.88	0.74	0.85	0.65	0.64	1.32	1.14
		150	Absolute value	16.23 ± 1.37	7.67 ± 1.79	23.89 ± 0.77	2.69 ± 0.34	0.53 ± 0.09	3.22 ± 0.30	1.81 ± 0.22	5.04 ± 0.58	0.17 ± 0.01	0.07 ± 0.01
			Relative value	0.49	0.50	0.49	0.80	0.62	0.76	0.52	0.52	1.65	1.26
		200	Absolute value	16.33 ± 3.23	5.66 ± 0.53	21.98 ± 3.43	2.63 ± 0.46	0.35 ± 0.06	2.98 ± 0.48	1.58 ± 0.13	3.39 ± 0.52	0.16 ± 0.01	0.06 ± 0.01
			Relative value	0.49	0.37	0.45	0.78	0.41	0.71	0.46	0.35	1.59	1.11
MST (3)	39	0	Absolute value	34.86 ± 1.57	19.80 ± 1.06	54.66 ± 1.88	3.95 ± 0.17	1.16 ± 0.09	5.11 ± 0.14	1.63 ± 0.13	14.35 ± 1.43	0.11 ± 2.21E-03	0.06 ± 1.61E-03
			Relative value	1.00	1.00	1.00	1.00	1.00	1.00	1.00	1.00	1.00	1.00
		50	Absolute value	35.92 ± 2.05	14.09 ± 2.34	50.01 ± 3.48	3.77 ± 0.20	0.89 ± 0.14	4.66 ± 0.29	1.63 ± 0.20	10.01 ± 1.47	0.11 ± 1.75E-03	0.06 ± 9.76E-04
			Relative value	1.03	0.71	0.91	0.95	0.77	0.91	1.00	0.70	0.93	1.08
		100	Absolute value	34.93 ± 2.55	8.99 ± 0.36	43.92 ± 2.44	3.67 ± 0.13	0.59 ± 0.07	4.27 ± 0.12	1.53 ± 0.16	8.65 ± 1.24	0.11 ± 4.26E-03	0.07 ± 4.65E-03
			Relative value	1.00	0.45	0.80	0.93	0.51	0.83	0.93	0.60	0.93	1.13
		150	Absolute value	24.46 ± 3.26	9.67 ± 0.35	34.13 ± 3.06	3.49 ± 0.35	0.64 ± 0.01	4.13 ± 0.35	1.63 ± 0.18	5.17 ± 0.98	0.14 ± 0.01	0.07 ± 3.23E-03
			Relative value	0.70	0.49	0.62	0.88	0.55	0.81	1.00	0.36	1.26	1.14
		200	Absolute value	21.76 ± 2.93	4.84 ± 0.71	26.61 ± 3.38	3.03 ± 0.13	0.37 ± 0.11	3.40 ± 0.15	1.39 ± 0.12	4.15 ± 0.61	0.14 ± 0.01	0.08 ± 0.01
			Relative value	0.62	0.24	0.49	0.77	0.32	0.66	0.85	0.29	1.24	1.29
	319	0	Absolute value	41.16 ± 1.57	19.29 ± 1.30	60.45 ± 2.29	4.23 ± 0.34	1.05 ± 0.12	5.28 ± 0.46	3.03 ± 0.24	10.10 ± 1.38	0.10 ± 0.01	0.05 ± 3.71E-03
			Relative value	1.00	1.00	1.00	1.00	1.00	1.00	1.00	1.00	1.00	1.00
		50	Absolute value	40.11 ± 2.47	14.81 ± 0.69	54.93 ± 3.10	3.81 ± 0.12	0.86 ± 0.08	4.67 ± 0.20	2.90 ± 0.26	8.42 ± 0.93	0.10 ± 3.40E-03	0.06 ± 2.92E-03
			Relative value	0.97	0.77	0.91	0.90	0.81	0.88	0.96	0.83	0.93	1.06
		100	Absolute value	29.49 ± 0.79	8.98 ± 0.98	38.47 ± 1.76	3.37 ± 0.25	0.60 ± 0.06	3.98 ± 0.30	2.69 ± 0.37	6.65 ± 0.57	0.11 ± 0.01	0.07 ± 1.21E-03
			Relative value	0.72	0.47	0.64	0.80	0.57	0.75	0.89	0.66	1.11	1.24
		150	Absolute value	26.32 ± 1.43	8.32 ± 1.26	34.64 ± 2.52	3.72 ± 0.06	0.54 ± 0.11	4.26 ± 0.09	2.17 ± 0.22	5.59 ± 0.81	0.14 ± 0.01	0.06 ± 4.72E-03
			Relative value	0.64	0.43	0.57	0.88	0.51	0.81	0.71	0.55	1.38	1.19
		200	Absolute value	20.71 ± 2.26	4.58 ± 0.18	25.29 ± 2.25	3.38 ± 0.48	0.38 ± 0.07	3.76 ± 0.42	2.28 ± 0.40	4.01 ± 0.65	0.16 ± 0.01	0.08 ± 0.01
			Relative value	0.50	0.24	0.42	0.80	0.36	0.71	0.75	0.40	1.58	1.54
	388	0	Absolute value	43.55 ± 1.20	21.85 ± 2.73	65.40 ± 3.86	4.14 ± 0.51	1.28 ± 0.17	5.42 ± 0.51	2.69 ± 0.27	9.73 ± 1.99	0.10 ± 0.01	0.06 ± 1.61E-03
			Relative value	1.00	1.00	1.00	1.00	1.00	1.00	1.00	1.00	1.00	1.00
		50	Absolute value	38.24 ± 0.42	11.87 ± 0.78	50.11 ± 0.78	3.92 ± 0.13	0.71 ± 0.12	4.63 ± 0.13	2.38 ± 0.38	7.89 ± 1.33	0.10 ± 2.45E-03	0.06 ± 0.01
			Relative value	0.88	0.54	0.77	0.95	0.55	0.85	0.89	0.81	1.08	1.01
		100	Absolute value	23.40 ± 1.65	11.42 ± 0.39	34.82 ± 2.04	3.47 ± 0.13	0.73 ± 0.07	4.20 ± 0.20	2.29 ± 0.18	6.21 ± 1.30	0.15 ± 0.01	0.06 ± 4.17E-03
			Relative value	0.54	0.52	0.53	0.84	0.57	0.77	0.85	0.64	1.56	1.09
		150	Absolute value	22.80 ± 2.55	8.13 ± 1.33	30.93 ± 2.70	3.37 ± 0.15	0.61 ± 0.10	3.98 ± 0.10	2.19 ± 0.25	5.15 ± 0.96	0.15 ± 0.01	0.07 ± 8.30E-04
			Relative value	0.52	0.37	0.47	0.81	0.47	0.73	0.82	0.53	1.56	1.28
		200	Absolute value	18.28 ± 1.38	6.40 ± 0.20	24.69 ± 1.27	2.81 ± 0.54	0.54 ± 0.03	3.35 ± 0.54	1.65 ± 0.23	3.67 ± 0.71	0.15 ± 0.02	0.08 ± 1.45E-03
			Relative value	0.42	0.29	0.38	0.68	0.42	0.62	0.62	0.38	1.61	1.45
	525	0	Absolute value	31.04 ± 1.02	19.46 ± 0.79	50.50 ± 1.24	2.97 ± 0.09	1.34 ± 0.20	4.31 ± 0.15	2.06 ± 0.30	12.18 ± 1.95	0.10 ± 3.07E-03	0.07 ± 0.01
			Relative value	1.00	1.00	1.00	1.00	1.00	1.00	1.00	1.00	1.00	1.00
		50	Absolute value	30.28 ± 1.07	14.13 ± 0.84	44.41 ± 1.86	3.19 ± 0.18	0.84 ± 0.07	4.03 ± 0.25	1.72 ± 0.18	9.34 ± 1.29	0.11 ± 3.73E-03	0.06 ± 1.66E-03
			Relative value	0.98	0.73	0.88	1.08	0.63	0.94	0.83	0.77	1.10	0.87

Supplementary Materials S1 continued

		100	Absolute value	23.15 ± 2.80	12.26 ± 1.39	35.41 ± 4.18	3.35 ± 0.25	0.69 ± 0.13	4.05 ± 0.38	1.99 ± 0.30	7.29 ± 1.21	0.15 ± 0.01	0.06 ± 0.01
			Relative value	0.75	0.63	0.70	1.13	0.52	0.94	0.97	0.60	1.52	0.82
		150	Absolute value	19.84 ± 0.94	10.61 ± 0.32	30.45 ± 1.25	3.10 ± 0.24	0.60 ± 0.08	3.70 ± 0.33	1.55 ± 0.18	6.17 ± 1.26	0.16 ± 0.01	0.06 ± 0.01
			Relative value	0.64	0.55	0.60	1.05	0.45	0.86	0.75	0.51	1.63	0.82
		200	Absolute value	/	4.38 ± 0.60	/	/	0.31 ± 0.03	/	/	4.73 ± 0.92	/	0.07 ± 2.19E-03
			Relative value	/	0.22	/	/	0.23	/	/	0.39	/	1.02
Average		0	Absolute value	37.65 ± 5.73	20.10 ± 1.19	57.75 ± 6.53	3.82 ± 0.58	1.21 ± 0.13	5.03 ± 0.50	2.35 ± 0.63	11.59 ± 2.13	0.10 ± 0.01	0.06 ± 0.01
			Relative value	1.00	1.00	1.00	1.00	1.00	1.00	1.00	1.00	1.00	1.00
		50	Absolute value	36.14 ± 4.26	13.73 ± 1.28	49.86 ± 4.30	3.67 ± 0.33	0.82 ± 0.08	4.50 ± 0.31	2.16 ± 0.60	8.92 ± 0.94	0.10 ± 4.73E-03	0.06 ± 2.38E-03
			Relative value	0.96	0.69	0.87	0.97	0.69	0.90	0.92	0.78	1.01	1.00
		100	Absolute value	27.74 ± 5.62	10.41 ± 1.68	38.15 ± 4.16	3.47 ± 0.15	0.66 ± 0.07	4.12 ± 0.13	2.13 ± 0.49	7.20 ± 1.07	0.13 ± 0.02	0.06 ± 4.93E-03
			Relative value	0.75	0.52	0.67	0.92	0.54	0.83	0.91	0.62	1.28	1.07
		150	Absolute value	23.36 ± 2.75	9.18 ± 1.18	32.54 ± 2.15	3.42 ± 0.26	0.60 ± 0.04	4.02 ± 0.24	1.89 ± 0.34	5.52 ± 0.48	0.15 ± 0.01	0.07 ± 0.01
			Relative value	0.63	0.46	0.57	0.91	0.50	0.80	0.82	0.49	1.46	1.10
		200	Absolute value	20.25 ± 1.78	5.05 ± 0.92	25.53 ± 0.98	3.07 ± 0.29	0.40 ± 0.10	3.50 ± 0.22	1.65 ± 0.45	4.14 ± 0.44	0.15 ± 0.01	0.08 ± 0.01
			Relative value	0.52	0.25	0.43	0.75	0.33	0.66	0.74	0.36	1.48	1.33
ST (4)	130	0	Absolute value	50.45 ± 4.57	20.95 ± 3.59	71.40 ± 8.03	3.84 ± 0.34	1.17 ± 0.26	5.01 ± 0.60	3.01 ± 0.29	12.85 ± 1.66	0.08 ± 3.36E-03	0.06 ± 3.99E-03
			Relative value	1.00	1.00	1.00	1.00	1.00	1.00	1.00	1.00	1.00	1.00
		50	Absolute value	34.30 ± 2.18	15.79 ± 1.22	50.09 ± 3.26	3.62 ± 0.16	0.93 ± 0.09	4.55 ± 0.23	2.36 ± 0.25	9.17 ± 1.18	0.11 ± 2.27E-03	0.06 ± 1.54E-03
			Relative value	0.68	0.75	0.70	0.94	0.79	0.91	0.78	0.71	1.39	1.06
		100	Absolute value	37.63 ± 0.77	11.03 ± 1.41	48.66 ± 2.07	3.87 ± 0.34	0.69 ± 0.14	4.55 ± 0.46	2.05 ± 0.25	7.48 ± 1.31	0.10 ± 0.01	0.06 ± 4.30E-03
			Relative value	0.75	0.53	0.68	1.01	0.59	0.91	0.68	0.58	1.35	1.11
		150	Absolute value	29.23 ± 3.08	9.25 ± 1.17	38.48 ± 3.05	3.43 ± 0.30	0.62 ± 0.12	4.05 ± 0.35	1.80 ± 0.35	6.18 ± 0.97	0.12 ± 2.28E-03	0.07 ± 0.01
			Relative value	0.58	0.44	0.54	0.89	0.53	0.81	0.60	0.48	1.54	1.21
		200	Absolute value	30.06 ± 2.48	6.37 ± 0.09	36.43 ± 2.42	3.33 ± 0.22	0.48 ± 0.06	3.81 ± 0.17	1.20 ± 0.16	3.64 ± 0.52	0.11 ± 2.80E-03	0.08 ± 0.01
			Relative value	0.60	0.30	0.51	0.87	0.41	0.76	0.40	0.28	1.45	1.36
	344	0	Absolute value	43.11 ± 2.55	21.32 ± 2.25	64.43 ± 2.57	4.03 ± 0.08	1.25 ± 0.11	5.28 ± 0.16	2.49 ± 0.72	10.89 ± 1.67	0.09 ± 3.79E-03	0.06 ± 3.91E-03
			Relative value	1.00	1.00	1.00	1.00	1.00	1.00	1.00	1.00	1.00	1.00
		50	Absolute value	39.44 ± 2.04	19.58 ± 1.29	59.02 ± 3.20	3.51 ± 0.08	1.13 ± 0.14	4.64 ± 0.15	2.32 ± 0.36	7.00 ± 1.21	0.09 ± 0.01	0.06 ± 3.93E-03
			Relative value	0.91	0.92	0.92	0.87	0.90	0.88	0.93	0.64	0.95	0.98
		100	Absolute value	33.52 ± 3.65	13.03 ± 2.24	46.54 ± 5.81	3.57 ± 0.46	0.79 ± 0.12	4.37 ± 0.56	2.06 ± 0.21	5.66 ± 1.14	0.11 ± 2.42E-03	0.06 ± 3.52E-03
			Relative value	0.78	0.61	0.72	0.89	0.63	0.83	0.83	0.52	1.14	1.04
		150	Absolute value	30.80 ± 3.92	7.68 ± 0.36	38.48 ± 4.23	3.56 ± 0.63	0.53 ± 0.09	4.09 ± 0.72	1.76 ± 0.35	5.37 ± 0.93	0.12 ± 0.01	0.07 ± 0.01
			Relative value	0.71	0.36	0.60	0.88	0.42	0.77	0.71	0.49	1.23	1.16
		200	Absolute value	24.82 ± 1.62	7.92 ± 0.43	32.74 ± 1.91	3.59 ± 0.02	0.48 ± 0.03	4.07 ± 0.01	1.11 ± 0.12	3.23 ± 0.64	0.14 ± 0.01	0.06 ± 8.73E-04
			Relative value	0.58	0.37	0.51	0.89	0.38	0.77	0.44	0.30	1.55	1.03
	527	0	Absolute value	45.11 ± 1.43	20.83 ± 1.28	65.94 ± 2.50	3.92 ± 0.14	1.14 ± 0.07	5.06 ± 0.20	3.32 ± 0.32	10.90 ± 2.37	0.09 ± 2.77E-03	0.05 ± 3.13E-03
			Relative value	1.00	1.00	1.00	1.00	1.00	1.00	1.00	1.00	1.00	1.00
		50	Absolute value	30.78 ± 2.86	19.49 ± 0.84	50.28 ± 2.97	3.42 ± 0.25	1.09 ± 0.07	4.51 ± 0.19	1.90 ± 0.40	9.69 ± 1.13	0.11 ± 0.02	0.06 ± 2.90E-03
			Relative value	0.68	0.94	0.76	0.87	0.95	0.89	0.57	0.89	1.29	1.02
		100	Absolute value	36.44 ± 1.55	10.90 ± 1.72	47.34 ± 3.16	3.74 ± 0.20	0.72 ± 0.11	4.46 ± 0.30	1.62 ± 0.26	8.08 ± 1.76	0.10 ± 3.25E-03	0.07 ± 0.01
			Relative value	0.81	0.52	0.72	0.95	0.63	0.88	0.49	0.74	1.18	1.21
		150	Absolute value	35.14 ± 1.86	7.52 ± 0.97	42.67 ± 1.45	3.72 ± 0.05	0.55 ± 0.06	4.27 ± 0.02	1.29 ± 0.21	6.21 ± 1.33	0.11 ± 0.01	0.07 ± 0.01
			Relative value	0.78	0.36	0.65	0.95	0.48	0.84	0.39	0.57	1.22	1.33
		200	Absolute value	19.41 ± 0.69	7.75 ± 0.85	27.15 ± 1.51	3.05 ± 0.26	0.52 ± 0.03	3.57 ± 0.29	0.95 ± 0.08	4.34 ± 1.47	0.16 ± 0.01	0.07 ± 3.70E-03
			Relative value	0.43	0.37	0.41	0.78	0.45	0.71	0.29	0.40	1.81	1.23

Supplementary Materials S1 continued

	667	0	Absolute value	41.10 ± 1.33	30.12 ± 1.50	71.22 ± 2.71	3.56 ± 0.09	1.68 ± 0.08	5.23 ± 0.09	2.66 ± 0.37	12.97 ± 1.40	0.09 ± 1.25E-03	0.06 ± 2.69E-03
			Relative value	1.00	1.00	1.00	1.00	1.00	1.00	1.00	1.00	1.00	1.00
		50	Absolute value	40.89 ± 1.41	16.82 ± 0.40	57.71 ± 1.80	4.20 ± 0.04	0.97 ± 0.05	5.17 ± 0.05	1.99 ± 0.37	10.73 ± 1.43	0.10 ± 2.87E-03	0.06 ± 2.50E-03
			Relative value	0.99	0.56	0.81	1.18	0.58	0.99	0.75	0.83	1.19	1.04
		100	Absolute value	34.24 ± 2.50	12.31 ± 0.90	46.55 ± 1.78	4.01 ± 0.18	0.72 ± 0.03	4.73 ± 0.15	1.73 ± 0.25	8.21 ± 1.43	0.12 ± 0.01	0.06 ± 4.15E-03
			Relative value	0.83	0.41	0.65	1.13	0.43	0.90	0.65	0.63	1.36	1.05
		150	Absolute value	36.22 ± 1.14	9.20 ± 1.32	45.42 ± 2.37	3.83 ± 0.18	0.64 ± 0.04	4.47 ± 0.15	1.59 ± 0.24	6.96 ± 1.27	0.11 ± 0.01	0.07 ± 0.01
			Relative value	0.88	0.31	0.64	1.08	0.38	0.85	0.60	0.54	1.22	1.26
		200	Absolute value	29.17 ± 1.40	5.37 ± 0.33	34.54 ± 1.44	3.58 ± 0.08	0.39 ± 0.04	3.97 ± 0.09	1.17 ± 0.12	4.71 ± 0.54	0.12 ± 3.53E-03	0.07 ± 4.12E-03
			Relative value	0.71	0.18	0.49	1.01	0.23	0.76	0.44	0.36	1.42	1.29
Average		0	Absolute value	44.94 ± 4.02	23.31 ± 4.55	68.25 ± 3.59	3.84 ± 0.20	1.31 ± 0.25	5.15 ± 0.13	2.87 ± 0.37	11.90 ± 1.16	0.09 ± 0.01	0.06 ± 1.79E-03
			Relative value	1.00	1.00	1.00	1.00	1.00	1.00	1.00	1.00	1.00	1.00
		50	Absolute value	36.35 ± 4.66	17.92 ± 1.91	54.27 ± 4.75	3.69 ± 0.35	1.03 ± 0.10	4.72 ± 0.31	2.14 ± 0.23	9.15 ± 1.57	0.10 ± 0.01	0.06 ± 1.13E-03
			Relative value	0.82	0.79	0.80	0.97	0.81	0.92	0.76	0.77	1.21	1.02
		100	Absolute value	35.46 ± 1.91	11.82 ± 1.03	47.27 ± 1.00	3.80 ± 0.19	0.73 ± 0.05	4.53 ± 0.15	1.87 ± 0.22	7.36 ± 1.17	0.11 ± 0.01	0.06 ± 3.35E-03
			Relative value	0.79	0.52	0.69	0.99	0.57	0.88	0.66	0.62	1.26	1.10
		150	Absolute value	32.85 ± 3.36	8.41 ± 0.94	41.26 ± 3.40	3.64 ± 0.18	0.59 ± 0.06	4.22 ± 0.19	1.61 ± 0.23	6.18 ± 0.65	0.11 ± 0.01	0.07 ± 2.69E-03
			Relative value	0.74	0.37	0.61	0.95	0.45	0.82	0.57	0.52	1.30	1.24
		200	Absolute value	25.87 ± 4.88	6.85 ± 1.21	32.72 ± 4.00	3.39 ± 0.26	0.47 ± 0.06	3.85 ± 0.22	1.11 ± 0.11	3.98 ± 0.67	0.13 ± 0.02	0.07 ± 0.01
			Relative value	0.58	0.31	0.48	0.89	0.37	0.75	0.39	0.34	1.56	1.22
HST (5)	137	0	Absolute value	51.20 ± 2.87	20.60 ± 2.24	71.80 ± 4.35	3.98 ± 0.29	1.06 ± 0.13	5.05 ± 0.35	4.74 ± 0.51	13.72 ± 2.22	0.08 ± 1.35E-03	0.05 ± 5.41E-04
			Relative value	1.00	1.00	1.00	1.00	1.00	1.00	1.00	1.00	1.00	1.00
		50	Absolute value	43.53 ± 0.53	16.07 ± 1.69	59.60 ± 1.78	3.68 ± 0.19	0.97 ± 0.09	4.65 ± 0.24	3.23 ± 0.51	11.06 ± 2.31	0.08 ± 3.25E-03	0.06 ± 2.49E-03
			Relative value	0.85	0.78	0.83	0.92	0.92	0.92	0.68	0.81	1.09	1.18
		100	Absolute value	40.79 ± 1.20	12.34 ± 1.91	53.13 ± 2.75	3.45 ± 0.11	0.77 ± 0.05	4.22 ± 0.13	2.83 ± 0.33	7.64 ± 1.59	0.08 ± 2.80E-04	0.06 ± 0.01
			Relative value	0.80	0.60	0.74	0.87	0.72	0.84	0.60	0.56	1.09	1.22
		150	Absolute value	40.70 ± 0.43	11.72 ± 1.40	52.42 ± 1.83	3.49 ± 0.18	0.74 ± 0.03	4.23 ± 0.21	2.19 ± 0.26	7.53 ± 1.19	0.09 ± 3.61E-03	0.06 ± 0.01
			Relative value	0.79	0.57	0.73	0.88	0.70	0.84	0.46	0.55	1.10	1.24
		200	Absolute value	39.87 ± 0.88	9.43 ± 1.27	49.29 ± 0.96	3.46 ± 0.16	0.64 ± 0.05	4.10 ± 0.18	1.69 ± 0.29	5.83 ± 0.96	0.09 ± 0.01	0.07 ± 0.01
			Relative value	0.78	0.46	0.69	0.87	0.60	0.81	0.36	0.43	1.12	1.32
	370	0	Absolute value	54.06 ± 3.49	20.77 ± 1.76	74.83 ± 5.17	3.95 ± 0.19	1.18 ± 0.04	5.13 ± 0.23	3.63 ± 0.37	8.81 ± 1.87	0.07 ± 2.10E-03	0.06 ± 3.02E-03
			Relative value	1.00	1.00	1.00	1.00	1.00	1.00	1.00	1.00	1.00	1.00
		50	Absolute value	45.03 ± 1.51	19.81 ± 1.73	64.84 ± 0.54	3.73 ± 0.19	1.16 ± 0.07	4.88 ± 0.19	2.40 ± 0.30	8.16 ± 1.69	0.08 ± 1.90E-03	0.06 ± 3.18E-03
			Relative value	0.83	0.95	0.87	0.94	0.98	0.95	0.66	0.93	1.13	1.03
		100	Absolute value	43.21 ± 1.91	15.61 ± 0.96	58.82 ± 2.86	3.65 ± 0.07	0.91 ± 0.01	4.56 ± 0.06	2.41 ± 0.31	6.81 ± 1.20	0.08 ± 2.44E-03	0.06 ± 4.24E-03
			Relative value	0.80	0.75	0.79	0.92	0.77	0.89	0.66	0.77	1.16	1.03
		150	Absolute value	38.78 ± 1.72	14.48 ± 1.07	53.26 ± 2.62	3.52 ± 0.21	0.86 ± 0.07	4.38 ± 0.28	1.90 ± 0.33	6.92 ± 0.84	0.09 ± 1.86E-03	0.06 ± 2.72E-03
			Relative value	0.72	0.70	0.71	0.89	0.73	0.85	0.52	0.79	1.24	1.05
		200	Absolute value	29.12 ± 2.58	10.96 ± 2.32	40.08 ± 3.78	3.29 ± 0.22	0.73 ± 0.16	4.02 ± 0.31	1.55 ± 0.20	5.81 ± 0.96	0.11 ± 2.45E-03	0.07 ± 3.32E-03
			Relative value	0.54	0.53	0.54	0.83	0.62	0.78	0.43	0.66	1.55	1.17
	460	0	Absolute value	54.09 ± 3.51	24.12 ± 1.33	78.22 ± 4.43	3.92 ± 0.28	1.52 ± 0.11	5.45 ± 0.38	2.45 ± 0.33	10.44 ± 1.11	0.07 ± 0.01	0.06 ± 1.04E-03
			Relative value	1.00	1.00	1.00	1.00	1.00	1.00	1.00	1.00	1.00	1.00
		50	Absolute value	40.95 ± 1.97	18.16 ± 1.84	59.10 ± 3.50	3.73 ± 0.29	1.07 ± 0.09	4.80 ± 0.36	1.75 ± 0.15	8.80 ± 0.70	0.09 ± 2.84E-03	0.06 ± 1.40E-03
			Relative value	0.76	0.75	0.76	0.95	0.70	0.88	0.72	0.84	1.25	0.94
		100	Absolute value	37.25 ± 2.78	13.77 ± 1.01	51.01 ± 3.36	3.79 ± 0.23	0.84 ± 0.09	4.63 ± 0.32	1.63 ± 0.12	6.72 ± 1.12	0.10 ± 3.11E-03	0.06 ± 2.86E-03
			Relative value	0.69	0.57	0.65	0.97	0.55	0.85	0.67	0.64	1.40	0.96

Supplementary Materials S1 continued

	150	Absolute value	36.85 ± 1.35	10.36 ± 1.03	47.21 ± 2.37	3.75 ± 0.12	0.67 ± 0.12	4.42 ± 0.23	1.25 ± 0.14	6.09 ± 0.53	0.10 ± 1.89E-03	0.06 ± 0.01
		Relative value	0.68	0.43	0.60	0.95	0.44	0.81	0.51	0.58	1.40	1.02
	200	Absolute value	30.39 ± 4.28	6.65 ± 0.15	37.04 ± 4.41	3.45 ± 0.38	0.44 ± 0.03	3.89 ± 0.40	1.12 ± 0.07	4.87 ± 0.72	0.11 ± 0.01	0.07 ± 3.44E-03
		Relative value	0.56	0.28	0.47	0.88	0.29	0.71	0.46	0.47	1.57	1.05
475	0	Absolute value	46.13 ± 0.31	28.73 ± 0.99	74.86 ± 1.02	4.03 ± 0.12	1.40 ± 0.12	5.43 ± 0.22	1.97 ± 0.30	12.72 ± 1.79	0.09 ± 2.30E-03	0.05 ± 2.68E-03
		Relative value	1.00	1.00	1.00	1.00	1.00	1.00	1.00	1.00	1.00	1.00
	50	Absolute value	42.68 ± 3.04	16.48 ± 0.69	59.15 ± 3.33	3.85 ± 0.22	0.93 ± 0.07	4.78 ± 0.17	1.53 ± 0.21	10.25 ± 1.56	0.09 ± 1.39E-03	0.06 ± 4.29E-03
		Relative value	0.93	0.57	0.79	0.96	0.67	0.88	0.78	0.81	1.03	1.16
	100	Absolute value	37.61 ± 0.73	18.52 ± 0.92	56.13 ± 1.16	3.48 ± 0.07	1.07 ± 0.08	4.56 ± 0.15	1.37 ± 0.28	6.97 ± 1.56	0.09 ± 3.47E-03	0.06 ± 0.01
		Relative value	0.82	0.64	0.75	0.86	0.77	0.84	0.70	0.55	1.06	1.19
	150	Absolute value	37.14 ± 0.45	10.55 ± 1.40	47.69 ± 1.59	3.48 ± 0.13	0.72 ± 0.03	4.20 ± 0.12	1.17 ± 0.11	6.53 ± 1.32	0.09 ± 4.20E-03	0.07 ± 0.01
		Relative value	0.81	0.37	0.64	0.87	0.51	0.77	0.60	0.51	1.07	1.41
	200	Absolute value	33.23 ± 1.35	5.01 ± 0.43	38.23 ± 1.33	3.66 ± 0.17	0.35 ± 0.07	4.01 ± 0.21	1.14 ± 0.11	6.00 ± 0.98	0.11 ± 0.01	0.07 ± 0.01
		Relative value	0.72	0.17	0.51	0.91	0.25	0.74	0.58	0.47	1.26	1.43
Average	0	Absolute value	51.37 ± 3.75	23.56 ± 3.81	74.93 ± 2.62	3.97 ± 0.05	1.29 ± 0.21	5.26 ± 0.20	3.20 ± 1.24	11.42 ± 2.22	0.08 ± 0.01	0.06 ± 0.01
		Relative value	1.00	1.00	1.00	1.00	1.00	1.00	1.00	1.00	1.00	1.00
	50	Absolute value	43.05 ± 1.70	17.63 ± 1.71	60.67 ± 2.79	3.75 ± 0.07	1.03 ± 0.10	4.78 ± 0.10	2.23 ± 0.76	9.57 ± 1.32	0.09 ± 4.09E-03	0.06 ± 1.66E-03
		Relative value	0.84	0.76	0.81	0.94	0.82	0.91	0.71	0.85	1.13	1.08
	100	Absolute value	39.71 ± 2.82	15.06 ± 2.67	54.77 ± 3.42	3.59 ± 0.16	0.90 ± 0.13	4.49 ± 0.18	2.06 ± 0.67	7.04 ± 0.42	0.09 ± 0.01	0.06 ± 2.11E-03
		Relative value	0.77	0.64	0.73	0.91	0.70	0.85	0.66	0.63	1.18	1.10
	150	Absolute value	38.37 ± 1.77	11.78 ± 1.90	50.15 ± 3.14	3.56 ± 0.13	0.75 ± 0.08	4.31 ± 0.11	1.63 ± 0.50	6.77 ± 0.61	0.09 ± 0.01	0.06 ± 3.66E-03
		Relative value	0.75	0.52	0.67	0.90	0.60	0.82	0.52	0.61	1.20	1.18
	200	Absolute value	33.15 ± 4.79	8.01 ± 2.68	41.16 ± 5.56	3.46 ± 0.15	0.54 ± 0.17	4.00 ± 0.09	1.37 ± 0.29	5.63 ± 0.51	0.11 ± 0.01	0.07 ± 1.65E-03
		Relative value	0.65	0.36	0.55	0.87	0.44	0.76	0.45	0.51	1.37	1.24

The data is the average ± SD, n = 4. / indicates no determination due to too high salt stress.
