# Supplementary material for: Root Na+ Content Negatively Correlated to Salt Tolerance Determines the Salt Tolerance of Brassica napus L. Inbred Seedlings
Source: Plants (Basel). 2022 Mar 29;11(7):906. doi: 10.3390/plants11070906 (PMC9002931; doi:10.3390/plants11070906)
Supplement: Supplementary file 1 [file plants-11-00906-s001.zip › Supplementary Materials S2.pdf]

Supplementary Materials S2. Ion contents and related indexes for the *B. napus* lines in the different salt-tolerance grades under different degrees of NaCl stress

Salt tolerance levels	No. of inbred lines	NaCl levels	ion content Na^+		K^+		Ca^{2+}		K^+/Na^+		Ca^{2+}/Na^+		$S_{K,Na}$	$S_{Ca,Na}$	S_{Na}	S_K	S_{Ca}
			Shoot (mmol/g)	Root（mmol/g)	Shoot (mmol/g)	Root（mmol/g)	Shoot (mmol/g)	Root（mmol/g)	Shoot	Root	Shoot	Root					
HSS (1)	557	0	0.63 ± 0.04	1.13 ± 0.08	0.10 ± 4.37E-03	0.24 ± 0.02	0.56 ± 0.03	0.69 ± 0.03	0.16 ± 0.02	0.21 ± 0.01	0.89 ± 0.10	0.62 ± 0.05	0.77 ± 0.07	1.46 ± 0.29	0.57 ± 0.07	0.44 ± 0.03	0.81 ± 0.06
		50	1.21 ± 0.06	1.48 ± 0.08	0.06 ± 0.01	0.17 ± 2.90E-03	0.48 ± 0.03	0.63 ± 0.01	0.05 ± 0.01	0.12 ± 0.01	0.39 ± 0.01	0.43 ± 0.01	0.43 ± 0.05	0.92 ± 0.04	0.82 ± 0.04	0.35 ± 0.04	0.75 ± 0.04
		100	1.66 ± 0.05	1.64 ± 0.04	0.04 ± 0.01	0.10 ± 0.01	0.39 ± 0.03	0.55 ± 0.01	0.02 ± 0.01	0.06 ± 0.01	0.24 ± 0.03	0.34 ± 0.01	0.40 ± 0.13	0.70 ± 0.10	1.02 ± 0.06	0.40 ± 0.12	0.71 ± 0.06
		150	2.24 ± 0.18	2.06 ± 0.16	0.04 ± 0.01	0.08 ± 0.01	0.37 ± 0.03	0.43 ± 0.02	0.02 ± 3.14E-03	0.04 ± 3.14E-03	0.16 ± 3.92E-03	0.21 ± 0.01	0.51 ± 0.04	0.79 ± 0.06	1.10 ± 0.17	0.56 ± 0.08	0.86 ± 0.09
		200	/	1.92 ± 0.14	/	0.05 ± 0.01	/	0.31 ± 0.04	/	0.03 ± 3.99E-03	/	0.16 ± 0.02	/	/	/	/	/
	611	0	0.58 ± 0.03	0.94 ± 0.08	0.13 ± 0.01	0.23 ± 0.02	0.62 ± 0.02	0.74 ± 0.01	0.22 ± 0.03	0.25 ± 0.03	1.06 ± 0.06	0.78 ± 0.06	0.89 ± 0.12	1.37 ± 0.18	0.62 ± 0.07	0.54 ± 0.02	0.84 ± 0.04
		50	1.13 ± 0.05	1.46 ± 0.05	0.09 ± 0.01	0.20 ± 0.02	0.55 ± 0.03	0.65 ± 0.06	0.08 ± 0.01	0.14 ± 0.02	0.49 ± 0.05	0.45 ± 0.04	0.59 ± 0.15	1.09 ± 0.05	0.77 ± 0.03	0.46 ± 0.10	0.85 ± 0.07
		100	1.56 ± 0.05	1.61 ± 0.04	0.07 ± 0.01	0.11 ± 0.01	0.49 ± 0.04	0.61 ± 0.02	0.04 ± 2.97E-03	0.07 ± 0.01	0.31 ± 0.02	0.38 ± 0.01	0.66 ± 0.03	0.83 ± 0.07	0.97 ± 0.03	0.64 ± 0.02	0.80 ± 0.08
		150	2.03 ± 0.14	1.88 ± 0.04	0.07 ± 2.90E-03	0.08 ± 3.16E-03	0.39 ± 0.01	0.50 ± 0.04	0.03 ± 3.29E-03	0.04 ± 2.02E-03	0.19 ± 0.02	0.26 ± 0.02	0.79 ± 0.04	0.73 ± 0.12	1.08 ± 0.05	0.85 ± 2.87E-03	0.78 ± 0.09
		200	2.13 ± 0.09	1.90 ± 0.16	0.04 ± 1.63E-03	0.06 ± 0.01	0.34 ± 0.02	0.36 ± 0.03	0.02 ± 1.62E-03	0.03 ± 0.01	0.16 ± 0.01	0.19 ± 0.02	0.64 ± 0.17	0.87 ± 0.18	1.12 ± 0.10	0.71 ± 0.13	0.96 ± 0.12
	668	0	0.61 ± 0.05	1.16 ± 0.05	0.10 ± 0.01	0.21 ± 0.03	0.53 ± 0.03	0.69 ± 0.02	0.17 ± 3.68E-03	0.18 ± 0.02	0.87 ± 0.03	0.60 ± 0.03	0.92 ± 0.13	1.45 ± 0.03	0.52 ± 0.02	0.48 ± 0.05	0.76 ± 0.03
		50	1.14 ± 0.03	1.52 ± 0.06	0.08 ± 0.01	0.19 ± 0.01	0.48 ± 0.04	0.65 ± 0.03	0.07 ± 4.07E-03	0.12 ± 0.01	0.42 ± 0.03	0.43 ± 0.03	0.56 ± 0.08	0.98 ± 0.14	0.75 ± 0.01	0.42 ± 0.06	0.74 ± 0.10
		100	1.52 ± 0.20	1.69 ± 0.13	0.05 ± 3.27E-03	0.08 ± 3.39E-03	0.42 ± 0.02	0.58 ± 0.02	0.04 ± 0.01	0.05 ± 2.80E-03	0.28 ± 0.04	0.34 ± 0.04	0.72 ± 0.12	0.81 ± 0.08	0.90 ± 0.08	0.64 ± 0.04	0.72 ± 5.37E-04
		150	2.22 ± 0.19	1.91 ± 0.13	0.05 ± 0.01	0.07 ± 4.31E-03	0.39 ± 0.02	0.44 ± 0.04	0.02 ± 2.15E-03	0.04 ± 3.97E-03	0.18 ± 0.02	0.23 ± 0.03	0.55 ± 0.11	0.77 ± 0.05	1.16 ± 0.05	0.64 ± 0.11	0.90 ± 0.08
		200	2.23 ± 0.15	1.89 ± 0.23	0.04 ± 0.01	0.07 ± 0.01	0.34 ± 0.04	0.37 ± 0.02	0.02 ± 2.45E-03	0.04 ± 0.01	0.15 ± 0.02	0.20 ± 0.03	0.56 ± 0.15	0.77 ± 0.09	1.19 ± 0.06	0.67 ± 0.18	0.91 ± 0.07
	703	0	0.56 ± 0.07	0.97 ± 0.05	0.09 ± 0.01	0.23 ± 0.02	0.58 ± 0.01	0.68 ± 0.04	0.16 ± 0.01	0.24 ± 0.02	1.06 ± 0.14	0.70 ± 0.03	0.70 ± 0.08	1.53 ± 0.26	0.57 ± 0.09	0.40 ± 0.08	0.86 ± 0.06
		50	1.07 ± 0.09	1.51 ± 0.04	0.06 ± 0.01	0.17 ± 0.01	0.51 ± 0.13	0.63 ± 0.01	0.06 ± 4.87E-03	0.12 ± 0.01	0.47 ± 0.09	0.42 ± 3.37E-03	0.52 ± 0.05	1.13 ± 0.21	0.71 ± 0.05	0.37 ± 0.05	0.81 ± 0.21
		100	1.63 ± 0.15	1.60 ± 0.06	0.06 ± 1.08E-03	0.09 ± 0.02	0.45 ± 0.05	0.57 ± 0.02	0.03 ± 3.71E-03	0.06 ± 0.01	0.28 ± 0.02	0.35 ± 0.02	0.62 ± 0.13	0.78 ± 0.09	1.02 ± 0.13	0.63 ± 0.12	0.79 ± 0.10
		150	1.91 ± 0.10	2.16 ± 0.17	0.05 ± 0.01	0.08 ± 0.01	0.37 ± 0.05	0.45 ± 0.04	0.03 ± 4.50E-03	0.04 ± 3.68E-03	0.19 ± 0.03	0.21 ± 0.03	0.81 ± 0.12	0.92 ± 0.07	0.89 ± 0.10	0.71 ± 0.05	0.82 ± 0.04
		200	2.08 ± 0.15	2.04 ± 0.08	0.04 ± 2.03E-03	0.07 ± 1.42E-03	0.33 ± 0.04	0.31 ± 0.02	0.02 ± 1.48E-03	0.03 ± 1.71E-03	0.16 ± 0.02	0.15 ± 0.02	0.57 ± 0.02	1.04 ± 0.12	1.02 ± 0.06	0.59 ± 0.04	1.07 ± 0.18
Average		0	0.59 ± 0.03	1.05 ± 0.11	0.11 ± 0.01	0.23 ± 0.01	0.57 ± 0.04	0.70 ± 0.03	0.18 ± 0.03	0.22 ± 0.03	0.97 ± 0.11	0.67 ± 0.08	0.82 ± 0.10	1.45 ± 0.07	0.57 ± 0.04	0.47 ± 0.06	0.82 ± 0.04
		50	1.14 ± 0.06	1.49 ± 0.03	0.07 ± 0.01	0.18 ± 0.01	0.51 ± 0.04	0.64 ± 0.01	0.06 ± 0.01	0.12 ± 0.01	0.44 ± 0.04	0.43 ± 0.01	0.53 ± 0.07	1.03 ± 0.10	0.76 ± 0.04	0.40 ± 0.05	0.79 ± 0.05
		100	1.59 ± 0.06	1.64 ± 0.04	0.05 ± 0.01	0.09 ± 0.01	0.44 ± 0.04	0.58 ± 0.02	0.03 ± 0.01	0.06 ± 0.01	0.28 ± 0.03	0.35 ± 0.02	0.60 ± 0.14	0.78 ± 0.05	0.98 ± 0.06	0.58 ± 0.12	0.76 ± 0.05
		150	2.10 ± 0.16	2.00 ± 0.13	0.05 ± 0.01	0.08 ± 1.70E-03	0.38 ± 0.01	0.45 ± 0.03	0.03 ± 0.01	0.04 ± 2.70E-03	0.18 ± 0.01	0.23 ± 0.03	0.66 ± 0.16	0.80 ± 0.08	1.06 ± 0.12	0.69 ± 0.12	0.84 ± 0.05
		200	2.15 ± 0.08	1.94 ± 0.07	0.04 ± 2.16E-03	0.06 ± 0.01	0.34 ± 4.32E-03	0.34 ± 0.03	0.02 ± 9.38E-04	0.03 ± 3.37E-03	0.16 ± 4.90E-03	0.17 ± 0.02	0.59 ± 0.04	0.89 ± 0.14	1.11 ± 0.08	0.65 ± 0.06	0.98 ± 0.08
SS (2)	40	0	0.63 ± 0.01	1.09 ± 0.11	0.12 ± 0.01	0.23 ± 0.01	0.56 ± 0.03	0.74 ± 0.02	0.18 ± 0.01	0.21 ± 0.01	0.89 ± 0.06	0.68 ± 0.06	0.88 ± 0.07	1.32 ± 0.13	0.58 ± 0.06	0.51 ± 0.07	0.76 ± 0.05
		50	1.14 ± 0.04	1.48 ± 4.60E-03	0.06 ± 4.81E-03	0.19 ± 0.01	0.47 ± 0.03	0.67 ± 0.02	0.06 ± 0.01	0.13 ± 0.01	0.42 ± 0.04	0.45 ± 0.01	0.44 ± 0.02	0.92 ± 0.09	0.77 ± 0.03	0.33 ± 3.21E-03	0.70 ± 0.05
		100	1.54 ± 0.08	1.58 ± 0.03	0.06 ± 0.01	0.11 ± 0.07	0.48 ± 0.06	0.60 ± 0.03	0.04 ± 3.53E-03	0.07 ± 0.05	0.31 ± 0.04	0.38 ± 0.02	0.71 ± 0.38	0.82 ± 0.07	0.98 ± 0.07	0.69 ± 0.38	0.80 ± 0.06
		150	1.86 ± 0.07	1.72 ± 0.01	0.05 ± 0.01	0.09 ± 0.02	0.42 ± 0.02	0.45 ± 0.03	0.03 ± 0.01	0.05 ± 0.01	0.23 ± 0.01	0.26 ± 0.02	0.54 ± 0.20	0.87 ± 0.03	1.08 ± 0.04	0.58 ± 0.20	0.94 ± 0.03
		200	1.94 ± 0.10	1.76 ± 0.04	0.05 ± 0.01	0.07 ± 0.01	0.35 ± 0.02	0.39 ± 0.01	0.02 ± 3.55E-03	0.04 ± 2.31E-03	0.18 ± 0.01	0.22 ± 0.01	0.59 ± 0.09	0.82 ± 0.05	1.10 ± 0.07	0.65 ± 0.10	0.90 ± 0.04
	74	0	0.51 ± 0.03	0.96 ± 0.03	0.11 ± 0.01	0.25 ± 0.02	0.58 ± 0.01	0.71 ± 0.04	0.23 ± 0.01	0.26 ± 0.03	1.15 ± 0.06	0.75 ± 0.05	0.86 ± 0.06	1.55 ± 0.19	0.53 ± 0.05	0.46 ± 0.03	0.82 ± 0.05
		50	1.25 ± 0.06	1.51 ± 0.01	0.06 ± 2.45E-03	0.18 ± 0.01	0.48 ± 0.05	0.65 ± 0.02	0.05 ± 7.46E-04	0.12 ± 0.01	0.38 ± 0.02	0.43 ± 0.01	0.43 ± 0.03	0.89 ± 0.07	0.83 ± 0.05	0.35 ± 0.01	0.74 ± 0.08
		100	1.60 ± 0.07	1.56 ± 0.02	0.06 ± 0.01	0.10 ± 0.01	0.42 ± 0.05	0.59 ± 0.03	0.04 ± 3.29E-03	0.06 ± 0.01	0.26 ± 0.03	0.38 ± 0.02	0.59 ± 0.04	0.71 ± 0.12	1.02 ± 0.06	0.60 ± 0.01	0.72 ± 0.10
		150	2.19 ± 0.03	1.88 ± 0.08	0.06 ± 2.43E-03	0.08 ± 0.01	0.41 ± 0.06	0.47 ± 0.04	0.03 ± 7.13E-04	0.04 ± 0.01	0.19 ± 0.03	0.25 ± 0.03	0.64 ± 0.11	0.76 ± 0.22	1.17 ± 0.06	0.75 ± 0.14	0.88 ± 0.21
		200	2.05 ± 0.09	1.91 ± 0.09	0.05 ± 0.01	0.06 ± 2.47E-03	0.31 ± 0.02	0.34 ± 0.02	0.02 ± 0.01	0.03 ± 2.53E-03	0.15 ± 0.01	0.18 ± 0.02	0.79 ± 0.11	0.85 ± 0.09	1.07 ± 0.09	0.85 ± 0.16	0.90 ± 0.05
	277	0	0.54 ± 0.05	1.09 ± 0.05	0.11 ± 0.03	0.26 ± 0.01	0.51 ± 0.01	0.71 ± 0.02	0.20 ± 0.05	0.24 ± 4.53E-03	0.96 ± 0.09	0.65 ± 0.03	0.85 ± 0.21	1.48 ± 0.19	0.50 ± 0.05	0.43 ± 0.14	0.72 ± 0.03
		50	1.00 ± 0.04	1.50 ± 0.09	0.08 ± 4.03E-03	0.19 ± 0.01	0.49 ± 0.02	0.67 ± 0.03	0.08 ± 0.01	0.13 ± 1.65E-03	0.49 ± 0.04	0.45 ± 0.04	0.61 ± 0.04	1.10 ± 0.04	0.66 ± 0.01	0.41 ± 0.03	0.73 ± 0.02
		100	1.46 ± 0.13	1.75 ± 0.16	0.06 ± 0.01	0.11 ± 4.48E-03	0.39 ± 0.03	0.62 ± 0.02	0.04 ± 0.01	0.06 ± 0.01	0.27 ± 0.02	0.36 ± 0.04	0.68 ± 0.09	0.75 ± 0.06	0.84 ± 0.02	0.57 ± 0.07	0.63 ± 0.05
		150	2.07 ± 0.23	2.08 ± 0.15	0.06 ± 3.85E-03	0.08 ± 0.01	0.36 ± 0.01	0.43 ± 0.04	0.03 ± 1.33E-03	0.04 ± 0.01	0.17 ± 0.02	0.21 ± 0.01	0.75 ± 0.16	0.84 ± 0.12	1.00 ± 0.12	0.74 ± 0.09	0.83 ± 0.06
		200	2.14 ± 0.10	2.02 ± 0.18	0.05 ± 0.01	0.06 ± 0.01	0.33 ± 0.01	0.37 ± 0.02	0.02 ± 0.01	0.03 ± 3.44E-03	0.15 ± 0.01	0.18 ± 0.02	0.78 ± 0.26	0.83 ± 0.02	1.06 ± 0.06	0.83 ± 0.29	0.88 ± 0.03
	323	0	0.58 ± 0.07	1.09 ± 0.15	0.11 ± 0.01	0.22 ± 0.03	0.56 ± 0.05	0.67 ± 0.05	0.19 ± 0.03	0.20 ± 1.43E-03	0.97 ± 0.05	0.62 ± 0.05	0.95 ± 0.15	1.57 ± 0.11	0.53 ± 0.07	0.51 ± 0.10	0.84 ± 0.08
		50	1.04 ± 0.08	1.54 ± 0.09	0.09 ± 2.44E-03	0.20 ± 0.02	0.54 ± 0.05	0.63 ± 0.04	0.08 ± 3.73E-03	0.13 ± 0.02	0.51 ± 0.01	0.41 ± 0.03	0.65 ± 0.11	1.26 ± 0.09	0.68 ± 0.09	0.43 ± 0.04	0.85 ± 0.10
		100	1.65 ± 0.12	1.73 ± 0.08	0.05 ± 0.01	0.08 ± 0.01	0.38 ± 0.07	0.60 ± 0.01	0.03 ± 4.27E-03	0.05 ± 0.01	0.23 ± 0.03	0.35 ± 0.01	0.69 ± 0.02	0.66 ± 0.07	0.96 ± 0.07	0.66 ± 0.06	0.63 ± 0.11
		150	1.90 ± 0.21	1.71 ± 0.06	0.05 ± 0.01	0.06 ± 2.53E-03	0.36 ± 0.03	0.39 ± 0.02	0.03 ± 0.01	0.04 ± 2.70E-03	0.19 ± 0.01	0.23 ± 0.01	0.67 ± 0.20	0.83 ± 0.05	1.11 ± 0.11	0.74 ± 0.17	0.93 ± 0.11
		200	1.90 ± 0.04	1.79 ± 0.15	0.05 ± 4.03E-03	0.07 ± 0.01	0.34 ± 0.05	0.37 ± 0.03	0.03 ± 2.68E-03	0.04 ± 0.01	0.18 ± 0.03	0.21 ± 0.01	0.75 ± 0.19	0.85 ± 0.17	1.07 ± 0.10	0.79 ± 0.12	0.89 ± 0.09
Average		0	0.56 ± 0.05	1.06 ± 0.07	0.11 ± 3.83E-03	0.24 ± 0.02	0.55 ± 0.03	0.71 ± 0.03	0.20 ± 0.02	0.23 ± 0.03	0.99 ± 0.11	0.67 ± 0.05	0.89 ± 0.05	1.48 ± 0.11	0.53 ± 0.03	0.48 ± 0.04	0.78 ± 0.05

Supplementary Materials S2 continued

		50	1.11 ± 0.11	1.51 ± 0.03	0.07 ± 0.01	0.19 ± 0.01	0.49 ± 0.03	0.66 ± 0.02	0.07 ± 0.02	0.13 ± 0.01	0.45 ± 0.06	0.44 ± 0.02	0.53 ± 0.11	1.04 ± 0.17	0.73 ± 0.08	0.38 ± 0.05	0.76 ± 0.07
		100	1.56 ± 0.08	1.66 ± 0.10	0.06 ± 3.31E-03	0.10 ± 0.01	0.42 ± 0.05	0.60 ± 0.01	0.04 ± 4.06E-03	0.06 ± 0.01	0.27 ± 0.03	0.37 ± 0.02	0.67 ± 0.05	0.73 ± 0.07	0.95 ± 0.08	0.63 ± 0.06	0.69 ± 0.08
		150	2.01 ± 0.15	1.85 ± 0.17	0.05 ± 0.01	0.08 ± 0.01	0.39 ± 0.03	0.44 ± 0.03	0.03 ± 1.09E-03	0.04 ± 0.01	0.19 ± 0.02	0.24 ± 0.02	0.65 ± 0.09	0.83 ± 0.05	1.09 ± 0.07	0.70 ± 0.08	0.90 ± 0.05
		200	2.01 ± 0.11	1.87 ± 0.12	0.05 ± 2.62E-03	0.07 ± 0.01	0.33 ± 0.02	0.37 ± 0.02	0.03 ± 1.78E-03	0.04 ± 4.98E-03	0.17 ± 0.02	0.20 ± 0.02	0.73 ± 0.09	0.83 ± 0.02	1.08 ± 0.02	0.78 ± 0.09	0.89 ± 0.01
MST (3)	39	0	0.59 ± 0.02	1.05 ± 0.11	0.12 ± 0.02	0.23 ± 0.01	0.55 ± 0.01	0.72 ± 0.01	0.20 ± 0.04	0.22 ± 0.01	0.93 ± 0.04	0.69 ± 0.07	0.89 ± 0.11	1.36 ± 0.10	0.57 ± 0.04	0.51 ± 0.10	0.77 ± 0.01
		50	1.05 ± 0.04	1.32 ± 0.09	0.09 ± 2.04E-03	0.18 ± 0.02	0.54 ± 0.02	0.68 ± 0.02	0.08 ± 3.60E-03	0.14 ± 0.02	0.51 ± 0.01	0.52 ± 0.02	0.62 ± 0.08	0.99 ± 0.04	0.80 ± 0.03	0.49 ± 0.07	0.79 ± 0.01
		100	1.58 ± 0.09	1.53 ± 0.03	0.08 ± 4.34E-03	0.10 ± 0.02	0.50 ± 0.03	0.65 ± 0.05	0.05 ± 6.73E-04	0.07 ± 0.01	0.31 ± 0.01	0.42 ± 0.02	0.76 ± 0.10	0.74 ± 0.02	1.03 ± 0.07	0.79 ± 0.15	0.77 ± 0.08
		150	1.98 ± 0.18	1.89 ± 0.15	0.07 ± 3.73E-03	0.07 ± 2.58E-03	0.38 ± 0.03	0.48 ± 0.01	0.03 ± 2.64E-03	0.04 ± 1.87E-03	0.19 ± 0.02	0.25 ± 0.01	0.90 ± 0.04	0.76 ± 0.05	1.05 ± 0.03	0.95 ± 0.02	0.80 ± 0.07
		200	2.14 ± 0.12	1.75 ± 0.13	0.06 ± 4.83E-03	0.07 ± 4.37E-03	0.36 ± 0.10	0.41 ± 0.04	0.03 ± 1.59E-03	0.04 ± 1.32E-03	0.17 ± 0.05	0.24 ± 0.04	0.72 ± 0.05	0.74 ± 0.33	1.23 ± 0.14	0.88 ± 0.05	0.89 ± 0.31
	319	0	0.51 ± 0.05	0.91 ± 0.09	0.10 ± 0.01	0.22 ± 0.02	0.52 ± 0.02	0.73 ± 0.07	0.19 ± 0.03	0.24 ± 4.14E-03	1.03 ± 0.06	0.80 ± 0.06	0.79 ± 0.10	1.29 ± 0.10	0.56 ± 0.01	0.44 ± 0.05	0.72 ± 0.07
		50	1.01 ± 0.05	1.47 ± 0.10	0.09 ± 0.01	0.20 ± 0.01	0.51 ± 0.02	0.67 ± 0.02	0.09 ± 0.01	0.14 ± 0.02	0.50 ± 0.04	0.45 ± 0.03	0.64 ± 0.10	1.10 ± 0.11	0.69 ± 0.02	0.44 ± 0.06	0.76 ± 0.06
		100	1.50 ± 0.04	1.57 ± 0.04	0.07 ± 0.01	0.11 ± 0.02	0.49 ± 0.05	0.65 ± 0.02	0.05 ± 0.01	0.07 ± 0.01	0.33 ± 0.04	0.42 ± 0.01	0.74 ± 0.15	0.78 ± 0.13	0.95 ± 0.05	0.71 ± 0.13	0.74 ± 0.09
		150	2.14 ± 0.04	1.77 ± 0.14	0.06 ± 4.80E-03	0.08 ± 0.02	0.39 ± 2.64E-03	0.46 ± 0.04	0.03 ± 2.02E-03	0.05 ± 0.01	0.18 ± 3.13E-03	0.26 ± 0.02	0.63 ± 0.11	0.71 ± 0.06	1.22 ± 0.11	0.78 ± 0.20	0.86 ± 0.07
		200	1.96 ± 0.08	1.84 ± 0.04	0.07 ± 0.01	0.08 ± 0.01	0.36 ± 0.03	0.39 ± 0.03	0.03 ± 3.10E-03	0.04 ± 3.55E-03	0.18 ± 0.01	0.21 ± 0.02	0.80 ± 0.12	0.86 ± 0.08	1.07 ± 0.05	0.86 ± 0.16	0.92 ± 0.11
	388	0	0.54 ± 0.13	1.03 ± 0.18	0.10 ± 4.53E-03	0.22 ± 0.02	0.53 ± 0.02	0.71 ± 0.04	0.20 ± 0.04	0.22 ± 0.05	1.01 ± 0.17	0.70 ± 0.13	0.93 ± 0.07	1.44 ± 0.18	0.52 ± 0.04	0.49 ± 0.04	0.75 ± 0.05
		50	0.95 ± 0.11	1.42 ± 0.06	0.08 ± 0.01	0.18 ± 0.02	0.47 ± 0.01	0.67 ± 0.06	0.08 ± 9.70E-04	0.13 ± 0.01	0.50 ± 0.06	0.47 ± 0.04	0.65 ± 0.08	1.07 ± 0.21	0.67 ± 0.09	0.43 ± 0.11	0.70 ± 0.08
		100	1.49 ± 0.11	1.49 ± 0.14	0.08 ± 4.95E-03	0.09 ± 0.01	0.46 ± 0.04	0.63 ± 0.06	0.06 ± 0.01	0.06 ± 0.01	0.31 ± 0.03	0.42 ± 0.04	0.94 ± 0.13	0.72 ± 0.04	1.00 ± 0.05	0.94 ± 0.18	0.72 ± 0.04
		150	1.93 ± 0.15	1.76 ± 0.07	0.08 ± 2.80E-03	0.09 ± 0.01	0.41 ± 0.03	0.49 ± 0.01	0.04 ± 2.27E-03	0.05 ± 3.12E-03	0.21 ± 0.01	0.28 ± 0.01	0.83 ± 0.06	0.77 ± 0.04	1.10 ± 0.08	0.92 ± 0.11	0.85 ± 0.05
		200	2.07 ± 0.11	1.86 ± 0.09	0.07 ± 0.01	0.07 ± 0.01	0.38 ± 0.02	0.37 ± 0.02	0.03 ± 1.28E-03	0.04 ± 0.01	0.18 ± 0.02	0.20 ± 0.01	0.89 ± 0.17	0.93 ± 0.09	1.11 ± 0.04	0.99 ± 0.19	1.03 ± 0.07
	525	0	0.56 ± 0.06	0.94 ± 0.11	0.11 ± 0.01	0.23 ± 0.01	0.56 ± 0.03	0.74 ± 0.04	0.20 ± 0.02	0.24 ± 0.02	0.99 ± 0.07	0.79 ± 0.05	0.82 ± 0.04	1.26 ± 0.12	0.60 ± 0.07	0.49 ± 0.05	0.75 ± 0.01
		50	1.13 ± 0.04	1.49 ± 0.06	0.07 ± 0.01	0.18 ± 0.02	0.49 ± 0.05	0.66 ± 0.03	0.06 ± 0.01	0.12 ± 0.02	0.43 ± 0.05	0.44 ± 0.03	0.53 ± 0.06	0.98 ± 0.14	0.76 ± 0.06	0.41 ± 0.05	0.74 ± 0.06
		100	1.52 ± 0.03	1.57 ± 0.02	0.07 ± 3.99E-03	0.10 ± 0.02	0.46 ± 0.04	0.58 ± 0.02	0.05 ± 2.10E-03	0.06 ± 0.01	0.30 ± 0.03	0.37 ± 0.01	0.74 ± 0.15	0.81 ± 0.12	0.97 ± 0.03	0.72 ± 0.17	0.79 ± 0.10
		150	1.89 ± 0.09	1.63 ± 0.09	0.06 ± 4.82E-03	0.09 ± 0.01	0.41 ± 0.03	0.47 ± 0.03	0.03 ± 2.17E-03	0.05 ± 4.25E-03	0.22 ± 0.01	0.29 ± 0.02	0.55 ± 0.06	0.75 ± 0.08	1.16 ± 0.11	0.63 ± 0.08	0.87 ± 0.11
		200	/	1.74 ± 0.09	/	0.07 ± 0.01	/	0.36 ± 0.06	/	0.04 ± 0.01	/	0.21 ± 0.03	/	/	/	/	/
Average		0	0.55 ± 0.03	0.98 ± 0.07	0.11 ± 0.01	0.22 ± 0.01	0.54 ± 0.02	0.72 ± 0.01	0.20 ± 3.92E-03	0.23 ± 0.01	0.99 ± 0.04	0.74 ± 0.06	0.86 ± 0.06	1.34 ± 0.08	0.56 ± 0.03	0.48 ± 0.03	0.75 ± 0.02
		50	1.04 ± 0.08	1.43 ± 0.08	0.08 ± 0.01	0.19 ± 0.01	0.50 ± 0.03	0.67 ± 0.01	0.08 ± 0.01	0.13 ± 0.01	0.49 ± 0.04	0.47 ± 0.03	0.61 ± 0.05	1.04 ± 0.06	0.73 ± 0.06	0.44 ± 0.04	0.75 ± 0.04
		100	1.52 ± 0.04	1.54 ± 0.04	0.08 ± 0.01	0.10 ± 0.01	0.47 ± 0.02	0.63 ± 0.03	0.05 ± 4.07E-03	0.06 ± 3.74E-03	0.31 ± 0.01	0.41 ± 0.03	0.80 ± 0.10	0.77 ± 0.04	0.99 ± 0.03	0.79 ± 0.11	0.76 ± 0.03
		150	1.98 ± 0.11	1.76 ± 0.11	0.07 ± 0.01	0.08 ± 0.01	0.40 ± 0.02	0.47 ± 0.01	0.03 ± 0.01	0.05 ± 0.01	0.20 ± 0.02	0.27 ± 0.02	0.73 ± 0.17	0.75 ± 0.03	1.13 ± 0.07	0.82 ± 0.14	0.84 ± 0.03
		200	2.06 ± 0.09	1.80 ± 0.06	0.07 ± 0.01	0.07 ± 0.01	0.37 ± 0.01	0.38 ± 0.02	0.03 ± 3.53E-03	0.04 ± 1.85E-03	0.18 ± 0.01	0.21 ± 0.02	0.80 ± 0.08	0.84 ± 0.09	1.14 ± 0.09	0.91 ± 0.07	0.95 ± 0.07
ST (4)	130	0	0.58 ± 0.05	1.04 ± 0.08	0.11 ± 4.33E-03	0.24 ± 0.03	0.55 ± 0.04	0.69 ± 0.03	0.19 ± 0.02	0.23 ± 0.01	0.94 ± 0.11	0.67 ± 0.02	0.81 ± 0.06	1.41 ± 0.21	0.57 ± 0.07	0.45 ± 0.04	0.79 ± 0.03
		50	1.08 ± 0.06	1.34 ± 0.03	0.10 ± 3.68E-03	0.20 ± 0.01	0.57 ± 0.04	0.68 ± 0.04	0.09 ± 2.96E-03	0.15 ± 0.01	0.53 ± 0.07	0.51 ± 0.04	0.59 ± 0.05	1.04 ± 0.13	0.81 ± 0.05	0.48 ± 0.01	0.84 ± 0.05
		100	1.41 ± 0.08	1.49 ± 0.07	0.06 ± 4.22E-03	0.10 ± 0.03	0.46 ± 0.02	0.65 ± 0.11	0.04 ± 1.72E-03	0.07 ± 0.01	0.33 ± 0.02	0.44 ± 0.09	0.65 ± 0.11	0.77 ± 0.18	0.95 ± 0.09	0.62 ± 0.13	0.72 ± 0.11
		150	1.99 ± 0.06	1.64 ± 0.09	0.06 ± 0.01	0.08 ± 0.01	0.42 ± 0.02	0.48 ± 0.04	0.03 ± 3.73E-03	0.05 ± 0.01	0.21 ± 3.50E-03	0.30 ± 0.04	0.67 ± 0.10	0.72 ± 0.08	1.22 ± 0.10	0.81 ± 0.16	0.87 ± 0.03
		200	2.09 ± 0.08	1.67 ± 0.10	0.07 ± 2.36E-03	0.08 ± 0.01	0.35 ± 0.02	0.42 ± 0.01	0.03 ± 1.13E-03	0.05 ± 0.01	0.17 ± 2.82E-03	0.25 ± 0.02	0.74 ± 0.09	0.68 ± 0.07	1.25 ± 0.04	0.92 ± 0.09	0.85 ± 0.07
	344	0	0.55 ± 0.03	1.11 ± 0.09	0.13 ± 0.01	0.25 ± 0.02	0.56 ± 0.02	0.72 ± 0.01	0.23 ± 0.01	0.22 ± 3.27E-03	1.02 ± 0.07	0.65 ± 0.05	1.06 ± 0.07	1.57 ± 0.07	0.50 ± 0.03	0.52 ± 0.04	0.78 ± 0.03
		50	0.99 ± 0.04	1.41 ± 0.04	0.10 ± 2.36E-03	0.19 ± 0.03	0.52 ± 0.02	0.67 ± 0.03	0.10 ± 0.01	0.13 ± 0.02	0.53 ± 0.04	0.48 ± 0.02	0.73 ± 0.08	1.11 ± 0.12	0.70 ± 0.05	0.51 ± 0.06	0.78 ± 0.04
		100	1.50 ± 0.03	1.54 ± 0.05	0.07 ± 0.01	0.09 ± 0.01	0.49 ± 0.04	0.63 ± 0.01	0.04 ± 0.01	0.06 ± 0.01	0.32 ± 0.03	0.41 ± 0.01	0.79 ± 0.13	0.79 ± 0.08	0.98 ± 0.04	0.77 ± 0.10	0.77 ± 0.05
		150	1.86 ± 0.12	1.71 ± 0.02	0.07 ± 0.01	0.09 ± 0.01	0.44 ± 0.08	0.51 ± 0.04	0.04 ± 0.01	0.05 ± 4.01E-03	0.24 ± 0.05	0.30 ± 0.02	0.75 ± 0.15	0.79 ± 0.14	1.09 ± 0.07	0.81 ± 0.11	0.86 ± 0.09
		200	1.94 ± 0.02	1.75 ± 0.07	0.06 ± 2.56E-03	0.08 ± 0.01	0.36 ± 0.03	0.38 ± 4.61E-03	0.03 ± 1.55E-03	0.04 ± 4.42E-03	0.19 ± 0.01	0.22 ± 0.01	0.74 ± 0.10	0.86 ± 0.09	1.11 ± 0.04	0.83 ± 0.12	0.95 ± 0.08
	527	0	0.56 ± 0.04	1.01 ± 0.14	0.13 ± 0.02	0.21 ± 0.01	0.53 ± 0.02	0.71 ± 0.07	0.23 ± 0.05	0.21 ± 0.02	0.96 ± 0.11	0.70 ± 0.04	1.09 ± 0.31	1.38 ± 0.25	0.56 ± 0.10	0.59 ± 0.09	0.76 ± 0.07
		50	1.12 ± 0.12	1.29 ± 0.02	0.08 ± 3.18E-03	0.20 ± 0.02	0.53 ± 0.01	0.66 ± 0.05	0.08 ± 0.01	0.16 ± 0.02	0.48 ± 0.04	0.51 ± 0.04	0.48 ± 0.03	0.94 ± 0.11	0.86 ± 0.08	0.42 ± 0.05	0.80 ± 0.05
		100	1.58 ± 0.08	1.56 ± 0.08	0.08 ± 0.01	0.09 ± 0.01	0.45 ± 0.01	0.63 ± 0.02	0.05 ± 3.88E-03	0.05 ± 0.01	0.28 ± 0.02	0.41 ± 0.02	0.89 ± 0.04	0.70 ± 0.04	1.02 ± 0.02	0.90 ± 0.05	0.71 ± 0.03
		150	2.09 ± 0.06	1.71 ± 0.14	0.07 ± 2.45E-03	0.07 ± 4.25E-03	0.40 ± 0.01	0.47 ± 0.02	0.03 ± 6.61E-04	0.04 ± 2.78E-03	0.19 ± 2.18E-03	0.28 ± 0.03	0.77 ± 0.04	0.69 ± 0.08	1.23 ± 0.09	0.95 ± 0.04	0.85 ± 0.05
		200	1.82 ± 0.03	1.47 ± 0.07	0.06 ± 0.01	0.08 ± 0.01	0.38 ± 0.02	0.37 ± 0.02	0.03 ± 2.38E-03	0.05 ± 0.01	0.21 ± 0.02	0.25 ± 0.02	0.62 ± 0.12	0.83 ± 0.03	1.24 ± 0.05	0.76 ± 0.11	1.03 ± 0.07
	667	0	0.59 ± 0.02	0.97 ± 0.04	0.13 ± 3.80E-03	0.25 ± 0.01	0.58 ± 0.03	0.72 ± 0.01	0.21 ± 2.32E-03	0.26 ± 0.02	0.98 ± 0.01	0.74 ± 0.03	0.83 ± 0.05	1.31 ± 0.06	0.61 ± 0.01	0.51 ± 0.03	0.80 ± 0.03
		50	0.99 ± 0.03	1.45 ± 0.02	0.08 ± 1.22E-03	0.17 ± 0.01	0.47 ± 0.02	0.69 ± 0.04	0.08 ± 1.54E-03	0.12 ± 0.01	0.47 ± 0.01	0.47 ± 0.03	0.67 ± 0.06	1.00 ± 0.04	0.68 ± 0.04	0.45 ± 0.02	0.68 ± 0.03
		100	1.48 ± 0.02	1.53 ± 0.05	0.07 ± 0.01	0.09 ± 0.01	0.43 ± 0.02	0.67 ± 0.03	0.04 ± 3.52E-03	0.06 ± 0.01	0.29 ± 0.01	0.44 ± 0.03	0.74 ± 0.12	0.66 ± 0.02	0.97 ± 0.03	0.72 ± 0.12	0.64 ± 0.01
		150	1.73 ± 0.09	1.58 ± 0.15	0.06 ± 4.59E-03	0.09 ± 0.01	0.38 ± 0.02	0.49 ± 0.03	0.03 ± 1.19E-03	0.06 ± 3.00E-03	0.22 ± 1.81E-03	0.31 ± 0.01	0.60 ± 0.05	0.71 ± 0.03	1.10 ± 0.10	0.66 ± 0.10	0.78 ± 0.05
		200	1.88 ± 0.03	1.69 ± 0.25	0.06 ± 0.01	0.08 ± 0.01	0.36 ± 0.02	0.41 ± 0.03	0.03 ± 2.83E-03	0.05 ± 3.27E-03	0.19 ± 0.01	0.25 ± 0.04	0.63 ± 0.04	0.79 ± 0.19	1.13 ± 0.17	0.71 ± 0.11	0.87 ± 0.09
Average		0	0.57 ± 0.02	1.03 ± 0.06	0.12 ± 0.01	0.24 ± 0.02	0.55 ± 0.02	0.71 ± 0.01	0.22 ± 0.02	0.23 ± 0.02	0.97 ± 0.03	0.69 ± 0.04	0.95 ± 0.15	1.42 ± 0.11	0.56 ± 0.05	0.52 ± 0.06	0.78 ± 0.02
		50	1.04 ± 0.07	1.37 ± 0.07	0.09 ± 0.01	0.19 ± 0.02	0.52 ± 0.04	0.67 ± 0.01	0.09 ± 0.01	0.14 ± 0.02	0.50 ± 0.03	0.49 ± 0.02	0.62 ± 0.11	1.02 ± 0.07	0.76 ± 0.09	0.47 ± 0.04	0.78 ± 0.07

Supplementary Materials S2 continued

		100	1.49 ± 0.07	1.53 ± 0.03	0.07 ± 0.01	0.09 ± 0.01	0.46 ± 0.02	0.65 ± 0.02	0.05 ± 2.02E-03	0.06 ± 0.01	0.31 ± 0.02	0.42 ± 0.02	0.77 ± 0.10	0.73 ± 0.06	0.98 ± 0.03	0.75 ± 0.12	0.71 ± 0.06
		150	1.92 ± 0.16	1.66 ± 0.06	0.07 ± 0.01	0.08 ± 0.01	0.41 ± 0.03	0.49 ± 0.02	0.03 ± 3.62E-03	0.05 ± 0.01	0.22 ± 0.02	0.30 ± 0.01	0.70 ± 0.08	0.73 ± 0.04	1.16 ± 0.07	0.81 ± 0.12	0.84 ± 0.04
		200	1.93 ± 0.12	1.65 ± 0.12	0.06 ± 0.01	0.08 ± 1.98E-03	0.36 ± 0.01	0.40 ± 0.02	0.03 ± 1.65E-03	0.05 ± 3.70E-03	0.19 ± 0.02	0.24 ± 0.02	0.68 ± 0.07	0.79 ± 0.08	1.18 ± 0.07	0.80 ± 0.09	0.93 ± 0.08
HST (5)	137	0	0.57 ± 0.01	1.08 ± 0.09	0.16 ± 2.15E-03	0.23 ± 0.02	0.62 ± 0.02	0.74 ± 0.04	0.29 ± 0.01	0.22 ± 0.02	1.07 ± 0.05	0.70 ± 0.07	1.31 ± 0.11	1.55 ± 0.09	0.54 ± 0.04	0.70 ± 0.05	0.83 ± 0.03
		50	1.05 ± 0.05	1.23 ± 0.05	0.12 ± 0.01	0.20 ± 0.03	0.58 ± 0.02	0.72 ± 0.07	0.11 ± 0.01	0.17 ± 0.02	0.56 ± 0.01	0.59 ± 0.08	0.67 ± 0.07	0.96 ± 0.11	0.85 ± 0.07	0.58 ± 0.10	0.81 ± 0.06
		100	1.49 ± 0.01	1.29 ± 0.11	0.10 ± 0.01	0.13 ± 0.04	0.55 ± 0.04	0.57 ± 0.11	0.06 ± 4.28E-03	0.10 ± 0.02	0.37 ± 0.03	0.44 ± 0.05	0.66 ± 0.11	0.85 ± 0.08	1.15 ± 0.09	0.76 ± 0.15	0.98 ± 0.13
		150	1.90 ± 0.03	1.34 ± 0.06	0.09 ± 0.01	0.09 ± 0.01	0.46 ± 0.04	0.46 ± 0.03	0.05 ± 0.01	0.07 ± 0.01	0.24 ± 0.02	0.34 ± 0.04	0.74 ± 0.12	0.71 ± 0.11	1.42 ± 0.07	1.05 ± 0.19	1.01 ± 0.12
		200	1.99 ± 0.05	1.43 ± 0.05	0.09 ± 0.01	0.07 ± 0.01	0.39 ± 0.03	0.42 ± 0.01	0.05 ± 0.01	0.05 ± 0.01	0.19 ± 0.01	0.29 ± 0.02	0.97 ± 0.22	0.67 ± 0.01	1.39 ± 0.08	1.34 ± 0.27	0.93 ± 0.06
	370	0	0.57 ± 0.03	0.93 ± 0.08	0.13 ± 0.01	0.24 ± 0.01	0.58 ± 1.52E-03	0.70 ± 0.02	0.22 ± 0.02	0.26 ± 0.02	1.02 ± 0.05	0.75 ± 0.08	0.84 ± 0.04	1.37 ± 0.16	0.62 ± 0.05	0.52 ± 0.04	0.84 ± 0.03
		50	0.99 ± 0.03	1.12 ± 0.03	0.09 ± 4.22E-03	0.17 ± 0.01	0.56 ± 0.03	0.63 ± 0.04	0.09 ± 2.53E-03	0.15 ± 0.01	0.57 ± 0.04	0.57 ± 0.02	0.57 ± 0.04	1.00 ± 0.08	0.89 ± 0.04	0.51 ± 0.06	0.89 ± 0.08
		100	1.49 ± 0.05	1.24 ± 0.17	0.08 ± 0.01	0.10 ± 0.01	0.49 ± 0.01	0.59 ± 0.02	0.05 ± 4.39E-03	0.08 ± 0.02	0.33 ± 0.02	0.48 ± 0.08	0.68 ± 0.17	0.69 ± 0.08	1.22 ± 0.16	0.81 ± 0.11	0.83 ± 0.02
		150	1.95 ± 0.10	1.30 ± 0.08	0.08 ± 3.87E-03	0.08 ± 1.88E-03	0.43 ± 0.05	0.46 ± 0.02	0.04 ± 4.04E-03	0.06 ± 2.81E-03	0.22 ± 0.03	0.36 ± 0.02	0.67 ± 0.05	0.62 ± 0.11	1.51 ± 0.06	1.01 ± 0.07	0.93 ± 0.13
		200	2.09 ± 0.07	1.41 ± 0.11	0.07 ± 0.01	0.06 ± 0.01	0.38 ± 0.01	0.36 ± 0.03	0.03 ± 2.48E-03	0.05 ± 0.01	0.18 ± 3.15E-03	0.26 ± 0.03	0.74 ± 0.06	0.71 ± 0.08	1.48 ± 0.11	1.09 ± 0.04	1.05 ± 0.11
	460	0	0.63 ± 0.05	0.85 ± 0.14	0.12 ± 0.03	0.23 ± 0.01	0.55 ± 0.02	0.72 ± 0.05	0.19 ± 0.04	0.27 ± 0.03	0.88 ± 0.08	0.85 ± 0.09	0.72 ± 0.24	1.04 ± 0.11	0.75 ± 0.10	0.53 ± 0.10	0.77 ± 0.04
		50	1.17 ± 0.03	1.24 ± 0.06	0.07 ± 0.01	0.18 ± 0.01	0.47 ± 0.04	0.65 ± 0.04	0.06 ± 4.61E-03	0.15 ± 0.02	0.40 ± 0.03	0.52 ± 0.03	0.42 ± 0.04	0.78 ± 0.09	0.95 ± 0.07	0.40 ± 0.03	0.74 ± 0.12
		100	1.55 ± 0.03	1.46 ± 0.08	0.08 ± 0.01	0.11 ± 0.01	0.49 ± 0.05	0.64 ± 0.04	0.05 ± 0.01	0.07 ± 0.01	0.32 ± 0.03	0.44 ± 0.04	0.66 ± 0.06	0.72 ± 0.10	1.06 ± 0.08	0.70 ± 0.03	0.76 ± 0.06
		150	1.86 ± 0.06	1.59 ± 0.07	0.07 ± 4.18E-03	0.10 ± 0.01	0.38 ± 0.04	0.49 ± 0.04	0.04 ± 1.60E-03	0.06 ± 0.01	0.21 ± 0.02	0.31 ± 0.03	0.62 ± 0.13	0.68 ± 0.07	1.17 ± 0.05	0.73 ± 0.12	0.79 ± 0.07
		200	1.93 ± 0.16	1.61 ± 0.08	0.06 ± 0.01	0.08 ± 0.01	0.35 ± 0.04	0.38 ± 0.04	0.03 ± 2.31E-03	0.05 ± 2.75E-03	0.18 ± 0.01	0.23 ± 0.03	0.66 ± 0.08	0.77 ± 0.11	1.20 ± 0.14	0.80 ± 0.18	0.92 ± 0.03
	475	0	0.59 ± 0.05	1.10 ± 0.05	0.12 ± 4.84E-03	0.24 ± 0.01	0.58 ± 0.05	0.72 ± 0.02	0.20 ± 0.01	0.22 ± 2.35E-03	0.98 ± 0.05	0.66 ± 0.03	0.90 ± 0.07	1.49 ± 0.10	0.54 ± 0.03	0.48 ± 0.03	0.80 ± 0.09
		50	1.13 ± 0.06	1.32 ± 0.09	0.09 ± 3.06E-03	0.19 ± 0.01	0.49 ± 0.02	0.65 ± 0.03	0.08 ± 1.88E-03	0.14 ± 1.16E-03	0.44 ± 0.03	0.49 ± 0.01	0.56 ± 0.01	0.88 ± 0.08	0.86 ± 0.10	0.49 ± 0.04	0.76 ± 0.04
		100	1.54 ± 0.10	1.48 ± 0.02	0.09 ± 0.01	0.11 ± 2.81E-03	0.50 ± 0.05	0.63 ± 0.06	0.06 ± 0.01	0.07 ± 2.96E-03	0.33 ± 0.05	0.43 ± 0.04	0.75 ± 0.10	0.77 ± 0.11	1.04 ± 0.05	0.78 ± 0.07	0.80 ± 0.08
		150	2.05 ± 0.19	1.38 ± 0.18	0.08 ± 4.61E-03	0.07 ± 2.79E-03	0.43 ± 0.05	0.51 ± 0.03	0.04 ± 2.42E-03	0.05 ± 0.01	0.21 ± 0.02	0.37 ± 0.04	0.73 ± 0.13	0.57 ± 0.11	1.50 ± 0.22	1.08 ± 0.06	0.84 ± 0.10
		200	1.91 ± 0.01	1.58 ± 0.08	0.07 ± 4.64E-03	0.07 ± 4.62E-03	0.35 ± 0.02	0.37 ± 0.03	0.03 ± 2.63E-03	0.04 ± 3.70E-03	0.18 ± 0.01	0.23 ± 0.03	0.81 ± 0.07	0.80 ± 0.10	1.21 ± 0.06	0.97 ± 0.10	0.96 ± 0.11
Average		0	0.59 ± 0.02	0.99 ± 0.12	0.13 ± 0.02	0.24 ± 0.01	0.58 ± 0.03	0.72 ± 0.02	0.22 ± 0.04	0.24 ± 0.03	0.99 ± 0.08	0.74 ± 0.08	0.95 ± 0.25	1.36 ± 0.23	0.61 ± 0.10	0.56 ± 0.10	0.81 ± 0.03
		50	1.09 ± 0.08	1.23 ± 0.08	0.09 ± 0.02	0.19 ± 0.01	0.53 ± 0.05	0.66 ± 0.04	0.09 ± 0.02	0.15 ± 0.01	0.49 ± 0.08	0.54 ± 0.04	0.56 ± 0.10	0.91 ± 0.10	0.89 ± 0.04	0.49 ± 0.07	0.80 ± 0.07
		100	1.52 ± 0.03	1.37 ± 0.12	0.08 ± 0.01	0.11 ± 0.01	0.51 ± 0.03	0.61 ± 0.03	0.06 ± 0.01	0.08 ± 0.01	0.33 ± 0.02	0.45 ± 0.03	0.69 ± 0.05	0.75 ± 0.07	1.12 ± 0.08	0.76 ± 0.05	0.84 ± 0.09
		150	1.94 ± 0.08	1.40 ± 0.13	0.08 ± 0.01	0.08 ± 0.01	0.43 ± 0.03	0.48 ± 0.02	0.04 ± 4.54E-03	0.06 ± 4.84E-03	0.22 ± 0.02	0.35 ± 0.03	0.69 ± 0.05	0.64 ± 0.06	1.40 ± 0.15	0.97 ± 0.16	0.89 ± 0.10
		200	1.98 ± 0.08	1.51 ± 0.10	0.07 ± 0.01	0.07 ± 0.01	0.37 ± 0.02	0.38 ± 0.02	0.04 ± 0.01	0.05 ± 2.64E-03	0.19 ± 0.01	0.25 ± 0.03	0.79 ± 0.13	0.74 ± 0.06	1.32 ± 0.14	1.05 ± 0.23	0.97 ± 0.06

The data is the average calculated by the formula, presented as Mean ± SD, n = 4.
